# Real‐time Voltammetric Anion Sensing Under Flow**

**DOI:** 10.1002/chem.202103249

**Published:** 2021-10-27

**Authors:** Sophie C. Patrick, Robert Hein, Mohamed Sharafeldin, Xiaoxiong Li, Paul D. Beer, Jason J. Davis

**Affiliations:** ^1^ Department of Chemistry University of Oxford South Parks Road Oxford OX1 3QZ UK

**Keywords:** halogen bonding, ion sensing, microfluidic sensing, sensors, voltammetry

## Abstract

The development of real‐life applicable ion sensors, in particular those capable of repeat use and long‐term monitoring, remains a formidable challenge. Herein, we demonstrate, in a proof‐of‐concept, the real‐time voltammetric sensing of anions under continuous flow in a 3D‐printed microfluidic system. Electro‐active anion receptive halogen bonding (XB) and hydrogen bonding (HB) ferrocene‐isophthalamide‐(iodo)triazole films were employed as exemplary sensory interfaces. Upon exposure to anions, the cathodic perturbations of the ferrocene redox‐transducer are monitored by repeat square‐wave voltammetry (SWV) cycling and peak fitting of the voltammograms by a custom‐written MATLAB script. This enables the facile and automated data processing of thousands of SW scans and is associated with an over one order‐of‐magnitude improvement in limits of detection. In addition, this improved analysis enables tuning of the measurement parameters such that high temporal resolution can be achieved. More generally, this new flow methodology is extendable to a variety of other analytes, including cations, and presents an important step towards translation of voltammetric ion sensors from laboratory to real‐world applications.

## Graphical abstract

**Sensing with the flow**: A continuous, real‐time ion sensing methodology under flow, enabled by repeat voltammetry and accompanying data analysis, is reported for the first time, herein exemplified for halogen bonding and hydrogen bonding‐mediated anion sensing at electroactive ferrocene‐isophthalamide‐(iodo)triazole films. The novel methodology enables sensing of multiple anionic targets, as well as long‐term sensing over multiple hours with highly reproducible responses and minimal signal loss.

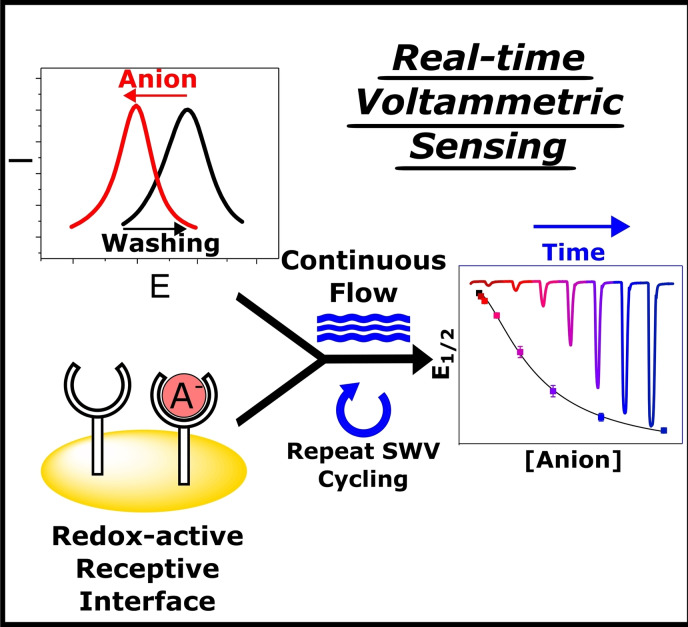

## Introduction

The real‐time, continuous sensing of ions is paramount in a wide variety of medical, environmental and technical scenarios, including long‐term health and water monitoring, but remains underdeveloped.^[1]^ Owing to their scalability, ease of use, high sensitivity and low cost, electrochemical ion sensing methodologies are ideally suited to address this challenge.^[2]^ However, to date, the only electroanalytical ion sensing approach with any significant real‐life impact are ion‐selective electrodes (ISEs).^[3]^ In order to widen this scope and to circumvent the drawbacks of ISEs, including thermodynamically limited sensitivities and comparably slow response times,^[4]^ other electroanalytical ion sensing methodologies are highly sought after.^[5]^ Of these, voltammetric ion sensors based on redox‐active synthetic supramolecular receptors are arguably the most prominent and are academically well‐established for the sensing of ions both in solution^[6]^ and at receptive interfaces.^[7]^ A notable advantage of these supramolecular redox sensors is an intimate binding transduction that is natively coupled to a (redox) modulation of ion binding strength. For example, the binding of anions can be significantly enhanced upon in situ electrochemical generation of an oxidised, cationic receptor state, thereby enabling anion binding, and sensing, in more polar, competitive (and real‐world‐relevant) solvents.[^2a^, ^7a^]

The interfacial voltammetric sensing of ions has, in particular, garnered significant attention in recent years,[^7a^, ^7b^, ^7c^, ^7e^, ^8^] carrying with it a number of advantages over solution‐phase sensing, including enhanced response magnitudes, removed solubility limitations, practical real‐world translation and sensor re‐use.[^7a^, ^9^] In spite of these advantages, the application of these sensors under real‐life relevant conditions remains unrealised. In order to achieve this ultimate goal two main challenges have to be addressed; the sensor needs to be able to operate continuously and provide a simple and calibratable ion‐specific response signal. The transient, non‐equilibrium nature of voltammetry (where responses are obtained by the analysis of the voltammograms) makes this a significant challenge. This is rarely of concern in academic studies, where the manual analysis of small data sets is not problematic, but is highly relevant to practical translation.

Herein, we address these challenges by reporting a novel methodology for continuous, interfacial voltammetric ion sensing through square‐wave voltammetry (SWV) and accompanying methods for analysing the acquired data sets. This enables the continuous, real‐time ion sensing under flow in a custom‐made 3D‐printed microfluidic cell, as exemplified for the sensing of various anions at recently reported, voltammetrically stable, halogen bonding (XB) and hydrogen bonding (HB) ferrocene‐isophthalamide‐(iodo)triazole receptive interfaces (**1.XB/HB_SAM_**). These findings present, to the best of our knowledge, the first example of continuous interfacial voltammetric ion sensing, paving the way for the translation of these systems towards practically applicable sensors.

## Results and Discussion

**Figure 1:**
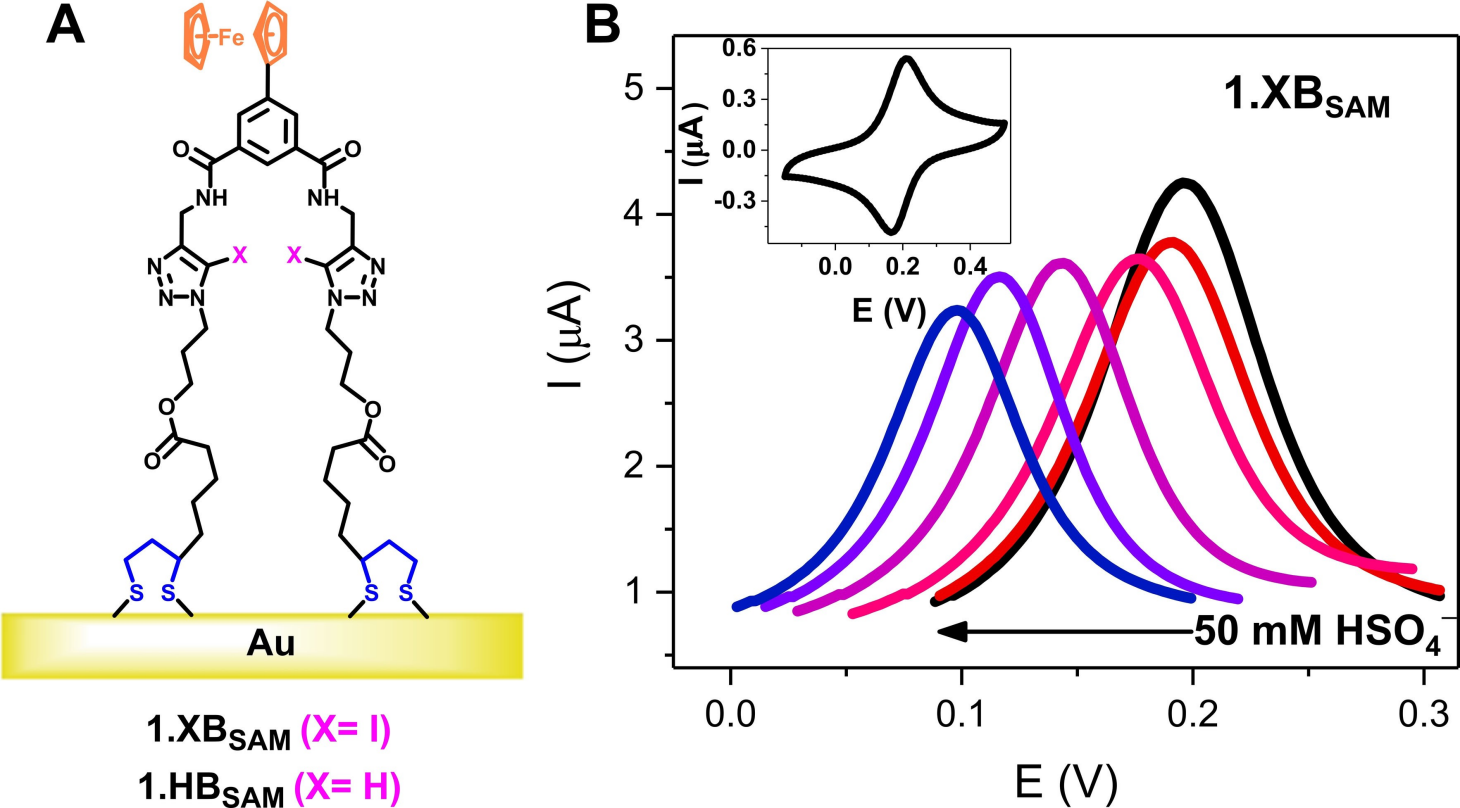
A) Schematic depiction of **1.XB/HB_SAM_** on gold electrode. B) SWVs showing cathodic voltammetric shift of **1.XB_SAM_** upon titration with HSO_4_^−^ up to 50 mM in ACN/H_2_O 99 : 1, 100 mM TBAClO_4_, 10 mM HClO_4_ (*E*_step_=2 mV, amplitude=20 mV, frequency=25 Hz). The inset shows the CV of **1.XB_SAM_** in the same solvent system at a scan rate of 0.1 V s^−1^.

The previously reported ferrocene‐isophthalamide‐(iodo)triazole SAMs **1.XB_SAM_** and **1.HB_SAM_** (shown in Figure 1A with iodo‐ and proto‐triazole binding sites, respectively) have been shown to be potent anion sensors. These reproducible, high‐density molecular films display significant anion binding induced cathodic (negative) voltammetric shifts of the ferrocene/ferrocenium (Fc/Fc^+^) redox couple upon exposure towards oxoanions and halides in the competitive ACN/H_2_O 99 : 1 solvent system as elucidated by square‐wave voltammetry upon sequential addition of anion aliquots to a static electrochemical cell (Figure 1B).^[7a]^ We noted that the acidification of the electrolyte with HClO_4_ significantly alleviated the otherwise significant redox signal loss of the interface upon repeat voltammetric cycling. This well‐known problem arises from reaction of ferrocenium (Fc^+^) with adventitious nucleophiles or electrolyte anions and can be significantly impeded by adding small amounts of an aqueous electrolyte of low pH, to protonate the basic nucleophiles and thus, scavenge them.^[10]^

**Figure 2:**
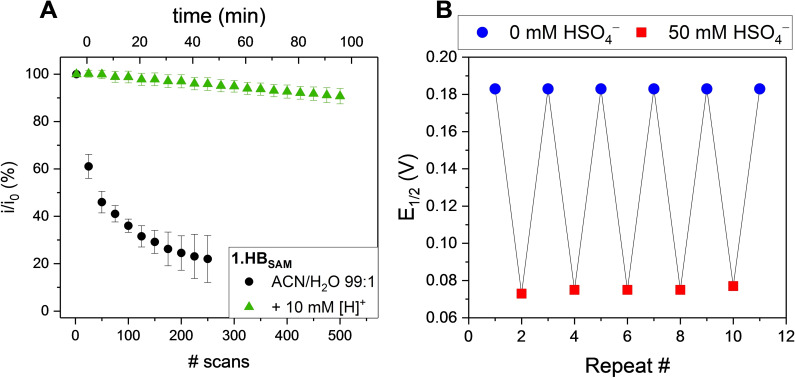
A) Redox signal stability, as assessed by the relative decrease in peak current *(* $i/i_{0}$ *)*, of **1.HB_SAM_** upon repeated SWV cycling in ACN/H_2_O 99 : 1, 100 mM TBAClO_4_ in the presence (green triangles) and absence (black circles) of 10 mM HClO_4_. The measurements in the absence of acid were terminated after 250 scans. The stability at intermediate acid concentrations is shown in Figure S2.1. B) Potential response of **1.HB_SAM_** in ACN/H_2_O 99 : 1, 10 mM HClO_4_ in the alternating absence and presence of 0 or 50 mM HSO_4_^−^.

As shown in Figure 2A, acidification has a profound effect on the redox stability (with a current retention of 99 % after 100 cycles and 91 % after 500 cycles for **1.HB_SAM_**), while signal loss in the absence of acid was much larger (only 36 % current retention after 100 cycles). This high voltammetric stability enables sensor re‐use with high signal integrity as shown in Figure 2B. Importantly, this is possible with minimal detriment to anion sensing, and is purely a specific means to circumvent the aforementioned stability issues of this specific interface. Further details regarding the effect of electrolyte acidification on the voltammetric characteristics and sensing capabilities of **1.XB/HB_SAM_** are detailed in the Supporting Information, Section S2.

While the response towards anions of low basicity (HSO_4_^−^ and Cl^−^) is not significantly affected by acidification, more basic anions such as H_2_PO_4_^−^ are protonated such that the voltammetric response is somewhat delayed but otherwise remains unchanged (Figures S2.2–S2.3). Of further note is that acidification only modulates the degree of anion protonation in solution and does not otherwise directly influence the response behaviour of the films.

Under these conditions we find that the binding/response isotherms are well‐described by the empirical Langmuir‐Freundlich model [Eq. (1)], from which the apparent anion binding constant *K*_app_ can be obtained (see Supporting Information Section S2 for more detailed discussions about the relevant binding equilibria).^[11]^

(1) $$\theta =\frac{{\left(K_{app}{}^{*}\left[A^{-}\right]\right)}^{n}}{1+{\left(K_{app}{}^{*}\left[A^{-}\right]\right)}^{n}}$$

To summarise thus far, we have demonstrated the capability of the redox‐active molecular films **1.XB/HB_SAM_** to reversibly recruit and respond to anions with high levels of signal stability in a standard (“static”) electrochemical set‐up. In the following we demonstrate, in a proof‐of‐principle, the application of these sensory interfaces for real‐time continuous flow anion sensing.

### Real‐time continuous flow anion sensing

**Figure 3:**
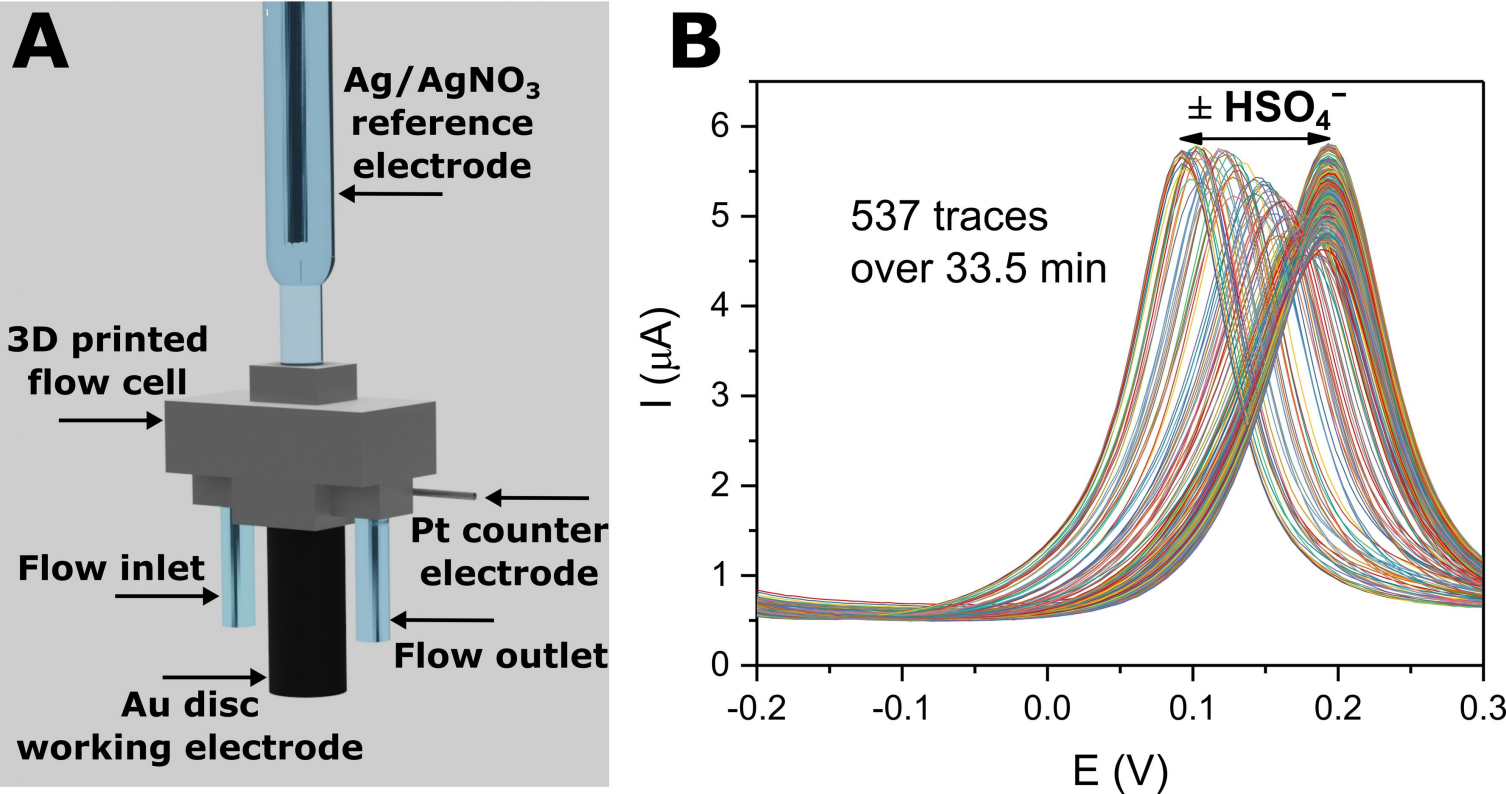
A) Schematic depiction of the 3D‐printed electrochemical flow cell. B) Overlay of all the recorded SWV traces afforded from a typical titration experiment (537 traces in 33.5 min), under continuous flow (*E*_step_=5 mV, amplitude=20 mV, frequency=50 Hz).

To this end, a custom 3D‐printed electrochemical flow cell with an internal chamber of 100 μL was designed (Figure 3A), through which the electrolyte (ACN/H_2_O 99 : 1, 100 mM TBAClO_4_, 10 mM HClO_4_) is continuously pumped with the help of a syringe pump (for further information see Figures S3.1–S3.3 and Supporting Information, Section S3). This flow cell was tolerant to a large range of flow rates (0.1–2 mL min^−1^) and displayed a stable, flow rate‐independent voltammetric response (see Figure S3.4). With the help of a sample injection valve system, aliquots of analyte solution of defined volume (typically 500 μL) were injected into the flow cell (at a flow rate of 500 μL min^−1^).^[12]^

**Figure 4:**
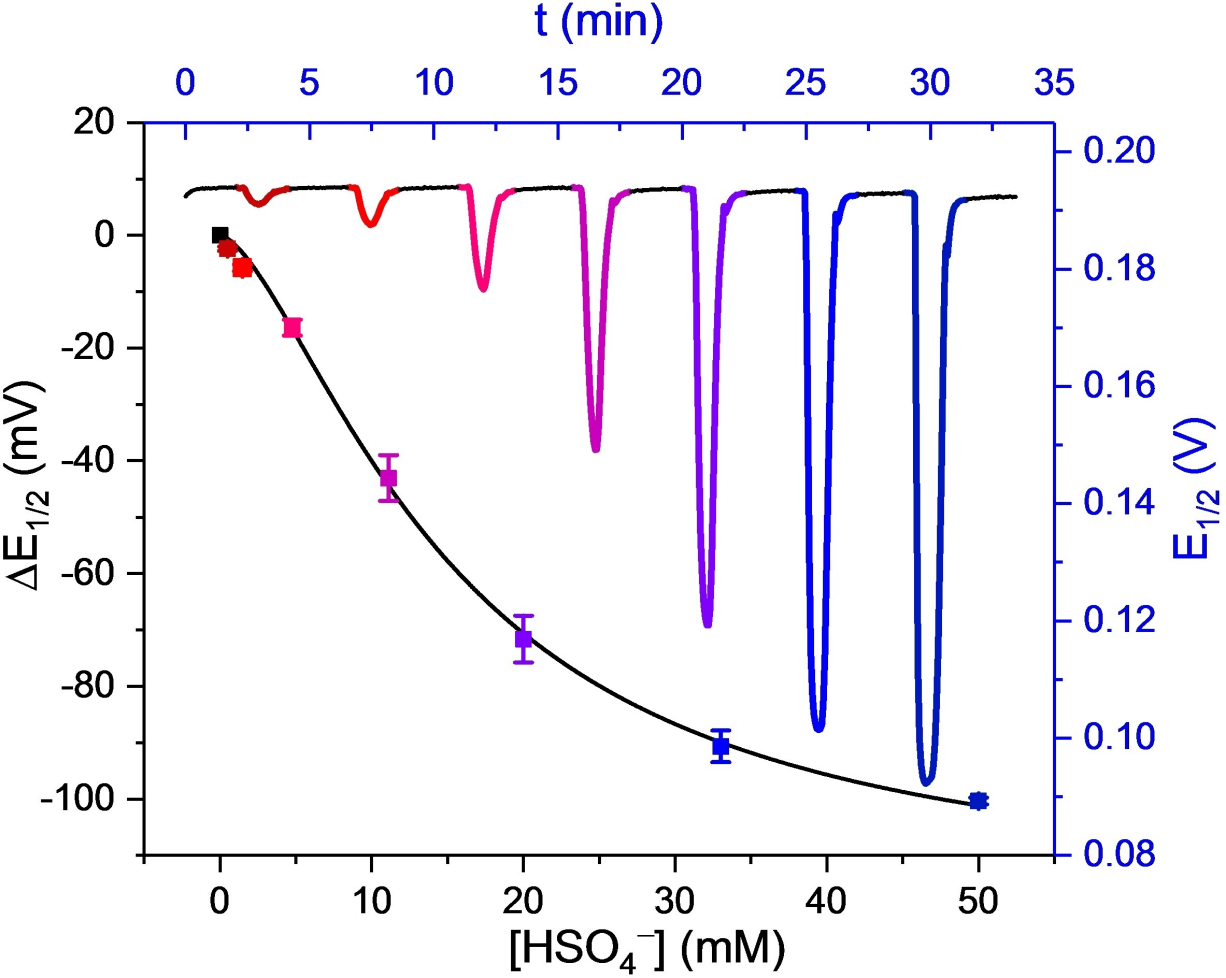
Sensogram (blue axes) and corresponding isotherm (black axes) from a typical titration experiment (Figure 3B) under continuous flow of **1.XB_SAM_** in response to increasing concentrations of HSO_4_^−^ in ACN/H_2_O 99 : 1 (100 mM TBAClO_4_, 10 mM HClO_4_). Each spike corresponds to injections of increasing [HSO_4_^−^] up to 50 mM. Data analysis of the voltammograms was carried out according to the AsymFit method as discussed in the Data Analysis Section below. The solid line represents a fit according to the Langmuir‐Freundlich model [Eq. (1)]. Error bars represent one standard deviation of independent triplicate measurements.

In analogy to standard, static conditions, anion sensing experiments were carried out by repeat SWV cycling, presenting both a comparably fast and straight‐forward way of probing the voltammetric properties of the sensor. Specifically, the sensors‘ half‐wave potential (*E*_1/2_), and its cathodic shift upon anion binding, can be readily obtained as the peak potential from the SW voltammograms (*E*_peak_). As expected, control injections of a non‐coordinating anion (20 mM PF_6_^−^) did not induce significant voltammetric responses (Figure S3.5), while HSO_4_^−^ induced notable cathodic response spikes, as shown in Figure 3B and Figure 4. Advantageously, as fresh electrolyte is continually flushed through the system, the injection of the analyte sample is immediately followed by a “washing” step with fresh electrolyte, such that the original potential baseline is re‐established.

The continuous SWV experiments were conducted with the optimised parameters of a step potential (*E*_step_) of 5 mV, an amplitude of 20 mV and a frequency (*f*) of 50 Hz over a potential range (*E*_range_) of 550 mV (−0.25–0.3 V). This window was chosen as it encompasses the original *E*_1/2_ (≈0.2 V) as well as cathodic perturbations of the Fc/Fc^+^ couple by up to −200 mV.

These parameters afforded, according to [Eq. (2)], a high temporal resolution of *t*_scan_ ≈3.7 s (see Supporting Information, Section S3.4 and Figure S3.6 for further details about parameter optimisation).

(2) $$t{}_{scan}=\;\frac{E_{range}}{f\;\times {\;E}_{step}}$$

**Figure 5:**
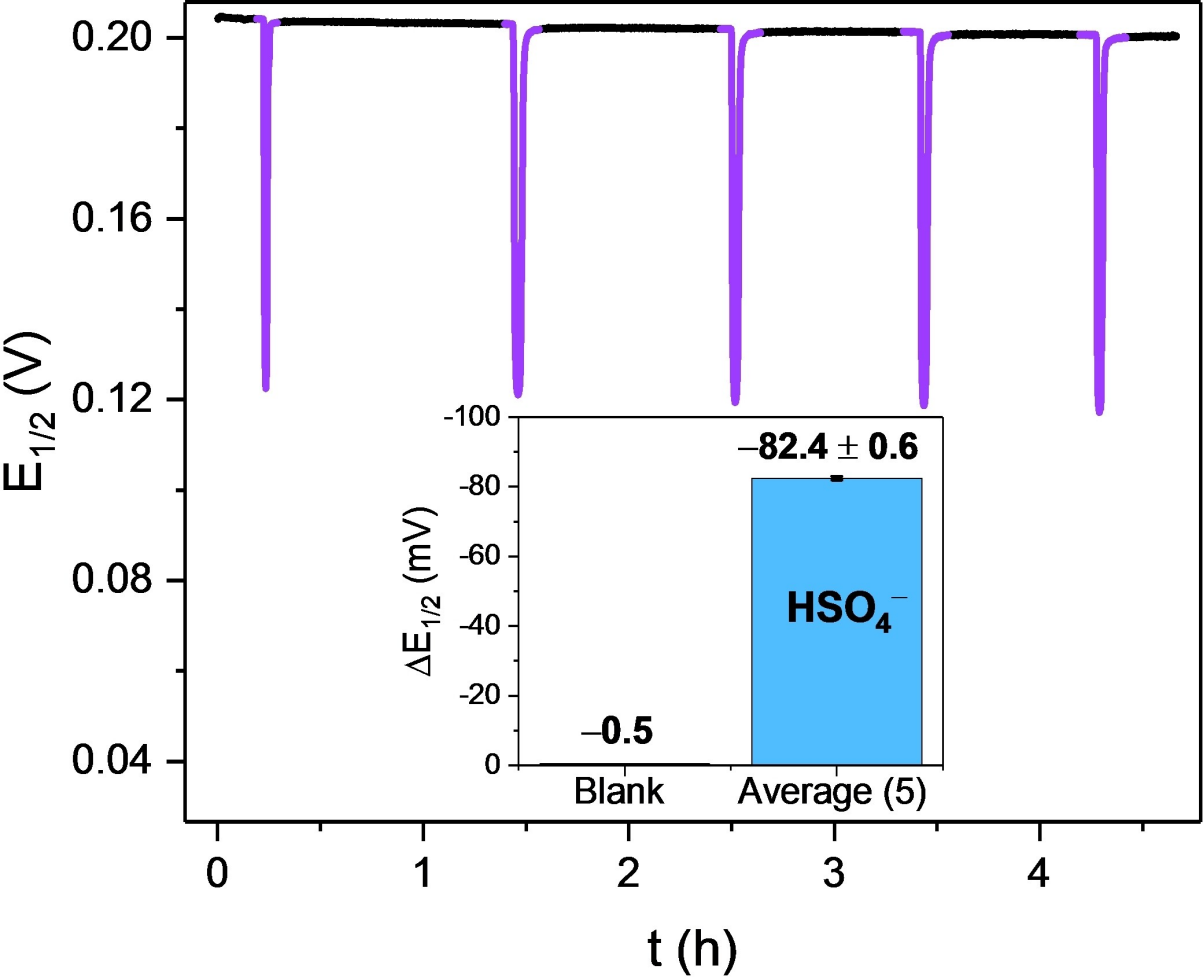
Voltammetric response of **1.XB_SAM_** towards five additions of HSO_4_^−^ under continuous electrolyte flow (flow rate=200 μL min^−1^) over 4.5 h. Each spike represents the response towards aliquots (*V*_Sample_=0.5 mL) of 20 mM HSO_4_^−^. The inset shows the voltammetric shift in response to the blank sample and the average of all five HSO_4_^−^ additions. Data analysis was carried out according to the AsymFit method.

As a result of the high voltammetric stability of the sensory interfaces, real‐time sensing over multiple hours is possible. As shown in Figure 5, **1.XB_SAM_** displayed a stable E_1/2_, with a drift of ≤5 mV over a continuous 4.5 h measurement (3700 scans). Repeat injections of 20 mM HSO_4_^−^ induced highly consistent response spikes with an average response magnitude of −82.4±0.6 mV. Although voltammetric (current) degradation (≈40 %) is inevitably observed over such a long‐term experiment (see Figure S3.7), this has no impact on either the *E*_1/2_ baseline or its responsiveness to anion recruitment, further highlighting the general utility of these interfaces as potential long‐term sensors.

### Data analysis

The accurate determination of the half‐wave potential *E*_1/2_ of the sensor interface is imperative to its sensing performance/accuracy. However, this parameter must be obtained by analysis of the voltammograms and is not directly available from the raw data, i. e. the voltammetric measurements do not afford a *direct* sensor (response) readout. For standard, static sensing experiments this analysis can easily be carried out manually, however, this is not feasible for continuous sensing applications in which a very high number of voltammograms are obtained. For example, in a typical continuous flow sensing experiment carried out herein more than 500 SWVs are recorded over ≈30 min (Figure 3B). We therefore developed MATLAB scripts (see Supporting Information, Section S4) to automate the analysis of raw voltammograms (i. e. to determine *E*_1/2_ for each trace) according to two different approaches: a simple data extraction (“PeakPick”) as well as a more advanced peak fitting (“AsymFit”) method.

**Figure 6:**
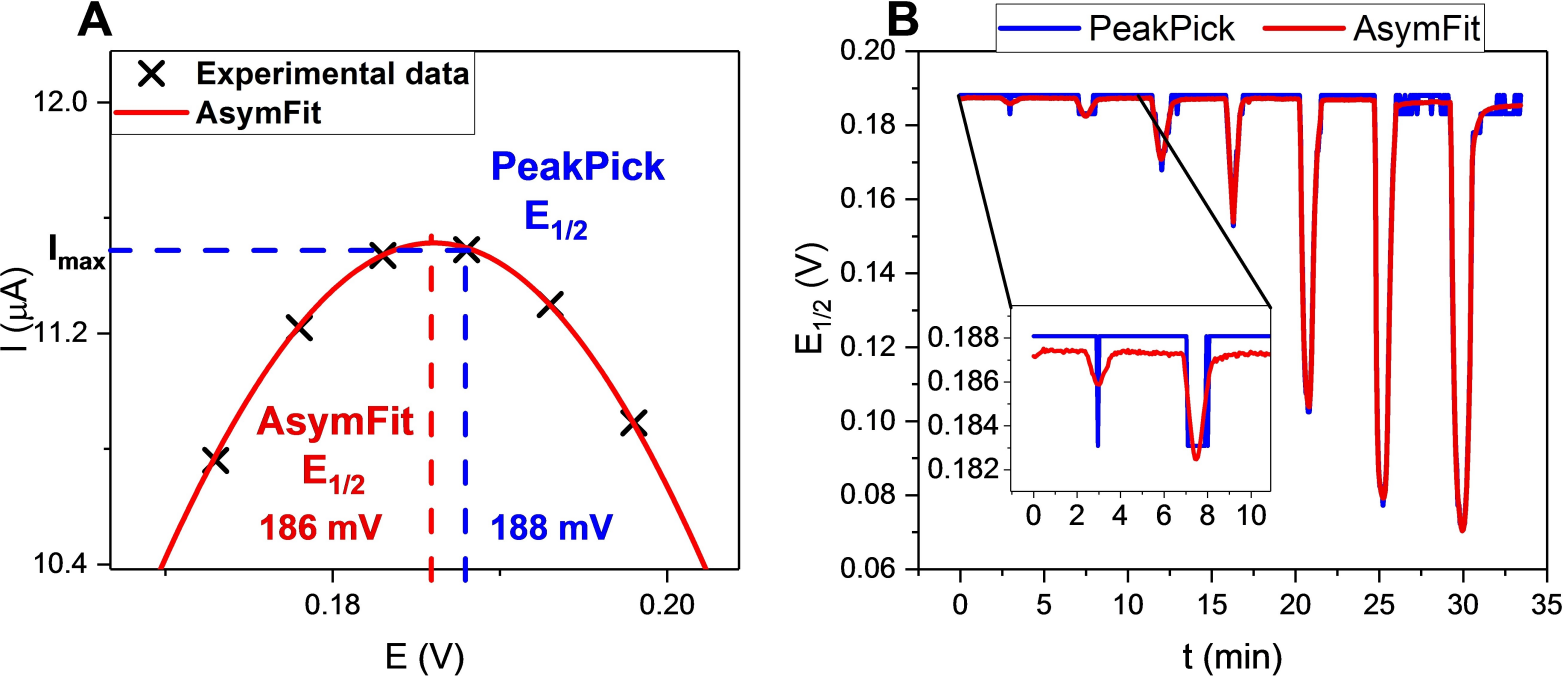
A) Schematic representation of *E*_1/2_ determination through the PeakPick and AsymFit methods. Real data points, recorded with *E*_step_=5 mV (amplitude=20 mV, frequency=50 Hz) are shown as black crosses with the fitted peak function [Eq. (S3)] shown as a red line. B) Comparison of sensograms of **1.HB_SAM_** in response to increasing concentrations of HSO_4_^−^ (0.5–50 mM) analysed with the PeakPick (black) and AsymFit (red) methods. The inset displays a magnification for the first two additions. As depicted, the *E*_1/2_ obtained by peak picking is significantly less accurate than that obtained by the AsymFit method.

*PeakPick Method*: The PeakPick method estimates *E*_1/2_ as the potential which corresponds to the peak maximum (maximum current, *I*_max_) of the recorded SWV (akin to peak detection functions in common electrochemical software), see Figure 6A and Supporting Information Section S4. While straightforward and easy to implement, the potential resolution of this method is inherently restricted to the magnitude of the step potential (5 mV herein). As shown in Figure 6A, this is associated with large errors in the determination of *E*_1/2_ and is predictably associated with a poor analytical performance (Figure 6B) and high limits of detection (LODs, see below). Additionally, this method is also more sensitive to noise which can induce further deviations from the true *E*_1/2_. Nevertheless, the PeakPick method serves as a useful starting point for more accurate data analysis through data fitting as described in the following.

*AsymFit Method*: To address the aforementioned limitations of the PeakPick approach, an improved method was devised whereby all SWV traces were fitted to an appropriate peak model from which a more accurate *E*_1/2_ can be determined. An asymmetric double sigmoidal model was selected [Eq. (S3)] as it provided excellent empirical fits to the data (Figure 6A, red line). Fitting of all voltammograms to this model was carried out with a custom MATLAB script, according to the following steps (visualised in Figure S4.1): An initial estimate for *E*_1/2_ was obtained via the PeakPick method. Subsequently all data points ±50 mV around this estimated value were isolated (i. e. the baseline was removed) and the peak fitted to [Eq. (S3)]. *E*_1/2_ was then extracted as the peak potential from this fitted, continuous peak fit.

**Figure 7:**
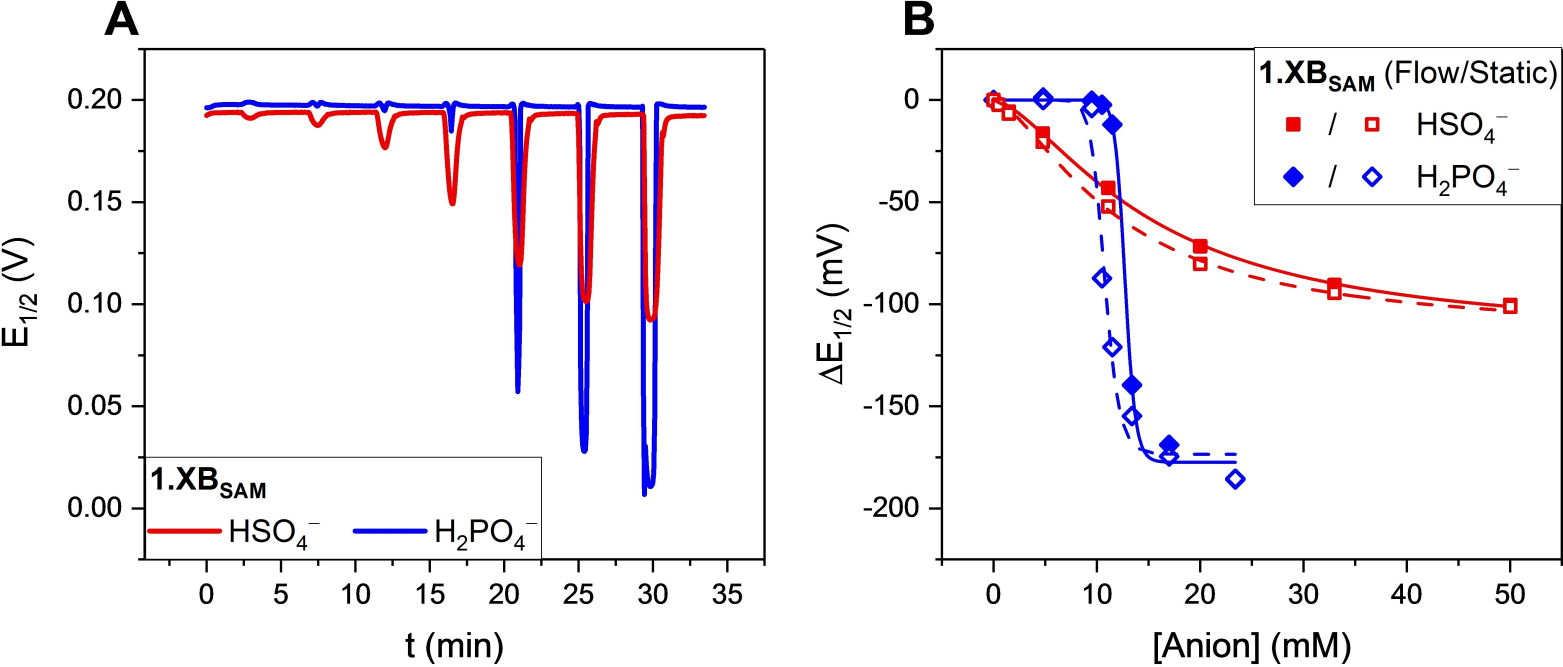
A) Sensograms of **1.XB_SAM_** in response to increasing concentrations of HSO_4_^−^ (red line) and H_2_PO_4_^−^ (blue line), analysed with the AsymFit method. B) Comparison of static (empty symbols) and continuous flow isotherms (filled symbols) of **1.XB_SAM_** in response to HSO_4_^−^ (red squares) and H_2_PO_4_^−^ (blue diamonds). Lines represent fits to the Langmuir‐Freundlich model [Eq. (1)]. The response towards H_2_PO_4_^−^ is “delayed” until ≈10 mM due to protonation. Error bars for HSO_4_^−^ are omitted here as they are too small to be resolved on this scale, see Figure S5.2.

This AsymFit method enables accurate determination of *E*_1/2_ even when the *E*_1/2_ falls in between two experimentally measured data points (where the PeakPick method is associated with significant error, Figure 6A) and thus affords sensograms with significantly higher potential‐resolution, as shown in Figures 4, 6B, 7 and Figures S4.2–S4.3. It is important to note that this methodology can be readily adapted to fit voltammetric data to any desired function, such as Gaussian or ab initio‐derived voltammetric models from which electrochemically relevant parameters, such as the full width at half maximum (FWHM) can be obtained.^**[13]**^ Similarly, simpler models can be employed to utilise less complex functions with less computational demand, allowing the user to tailor this methodology to their specific requirements.

### Sensing of other anions and analytical performance of the flow sensors

In order to demonstrate the general utility of these interfaces we carried out a series of continuous flow voltammetric anion sensing studies of both **1.XB/HB_SAM_** using the optimised experimental parameters and the AsymFit data analysis method. As shown in Figure 7A and Figures S5.1–S5.4, both interfaces responded sensitively to increasing concentrations of the oxoanions HSO_4_^−^ and H_2_PO_4_^−^ as well as Cl^−^, with response magnitudes that are nearly identical to those obtained under standard, static conditions (Figure 7B).^[7a]^ For example, under flow the cathodic shift of **1.XB_SAM_** in the presence of 50 mM HSO_4_^−^ was −100±1 mV which was found to be not only highly reproducible, but also identical to the response magnitudes under static conditions (−101±2 mV; Table 1). Similarly, the flow response isotherms (and their associated *K*_app_) as well as the sensitivity (slope of linear response regime) and LODs of the sensors were in good agreement with those obtained under static conditions (Table 1 and Tables S1–S8), again, confirming good analytical performance of the sensory interfaces under flow.^[14]^ For example, **1.HB_SAM_** displayed similar apparent binding constants, *K*_app_ of 75.3±4.1 vs. 67.9±3.0 M^−1^ in response to HSO_4_^−^, under static and continuous flow conditions, respectively.

**Table 1:** Maximum shifts Δ*E*_max_ (mV) obtained at the highest measured concentration for each anion, sensitivities (slope of linear region) and limits of detection (LOD) of **1.XB/HB_SAM_** under continuous flow (*E*_step_=5 mV) and under standard, static conditions (*E*_step_=2 mV).

Anion		Continuous Flow Voltammetry		Static Voltammetry	
		**1.XB_SAM_**	**1.HB_SAM_**	**1.XB_SAM_**	**1.HB_SAM_**
HSO_4−_	Δ*E*_max_ [mV]^[a]^	−100±1	−115±2	−101±2	−119±2
	Sensitivity [mV mM^−1^]^[b]^	4.08±0.13	3.84±0.73	4.68±0.13	5.26±0.09
	LOD [μM]	59.8±5.5	35.0±16.4	67.7±25.8	44.6±13.5
H_2_PO_4−_	Δ*E*_max_ [mV]^[c]^	−186	−189	−186	−203
	Sensitivity [mV mM^−1^]	49.9^[e]^	59.3^[e]^	36.0^[f]^	41.8^[f]^
	LOD [μM]^[d]^	3.77	0.894	20.1	8.58
Cl^−^	Δ*E*_max_ [mV]^[g]^	−83^[d]^	−77	−94	−88
	Sensitivity [mV mM^−1^]^[b]^	2.27	1.93	2.71	2.08
	LOD [μM]	89.6	48.0	210	195

Data analysis was carried out according to the AsymFit method. Errors, where shown, represent the average and one standard deviation of independent triplicate experiments. [a] At [HSO_4_^−^]=50 mM. [b] For range of 0–11 mM. [c] At [H_2_PO_4_^−^]=23.4 mM. [d] For binding isotherms that were corrected by −10 mM, see Figure S5.3 and associated discussions. [e] For range of 10.5–13.4 mM. [f] For range of 9.5–13.4 mM. [g] At [Cl^−^]=33 mM.

It is important to note that analyses via the simple PeakPick algorithm affords LODs that are significantly worse than those obtained from the AsymFit method, see Table 2. For example, even with the smaller, more accurate *E*_step_=2 mV, the PeakPick analysis affords a high LOD of 825±138 μM, for flow sensing of HSO_4_^−^ with **1.XB_SAM_**, 15‐fold worse than that of the AsymFit method (53.0±20.8 μM).^[15]^ This can be attributed to a significantly poorer signal‐to‐noise ratio (i. e. larger baseline fluctuation) obtained from the PeakPick methodology as its potential‐resolution is inherently limited to *E*_step_. Consequently, an improvement in LOD via PeakPick is only possible by significantly decreasing *E*_step_, which is, however, associated with a significant loss in temporal resolution [see Eq. (2)]. In contrast, the AsymFit analysis is not sensitive to the value of *E*_step_; the LOD for *E*_step_=5 mV is with 59.8±5.5 μM identical to that for *E*_step_=2 mV. These findings validate the choice of *E*_step_=5 mV as the chosen experimental parameter and saliently illustrates the value of the AsymFit method, not only enabling sensing with a significantly improved temporal resolution and a simple, automated signal readout, but also improved LODs.

**Table 2:** Comparison of LODs determined through PeakPick and AsymFit data analysis methods for **1.XB_SAM_** in response to HSO_4_^−^ under static conditions (*E*_step_=2 mV) and continuous flow (*E*_step_=2 or 5 mV).

**1.XB_SAM_**	Analysis Method	LOD [μM]
Flow	AsymFit, *E*_step_=5 mV	59.8±5.5
	Asymfit, *E*_step_=2 mV	53.0±20.8
	PeakPick, *E*_step_=2 mV	825±138
Static	AsymFit, *E*_step_=2 mV	67.7±25.8
	PeakPick, *E*_step_=2 mV	509±151

Errors represent one standard deviation of triplicate independent measurements.

Due to its high temporal resolution (≈3.7 s), this flow (+AsymFit) methodology may also serve to elucidate interfacial binding kinetics. For example, the Cl^−^ (de)complexation was observed to be significantly slower than those of the two oxoanions, an unexpected, yet potentially a profound observation in the study of interfacial ion recognition. Specifically, under the standard flow measurement conditions (flow rate of 500 μL min^−1^ and analyte volume of 500 μL) the response magnitude of both **1.XB_SAM_** and **1.HB_SAM_** towards chloride was significantly lower than under static conditions (Figures S5.4 and S5.5), attributable to insufficient exposure time of this analyte to the interface. Increasing the sample injection volume to 1 mL (and thus a doubling in exposure time) was sufficient to reach the static, equilibrium response.

## Conclusion

In summary, we herein present the first proof‐of‐principle example of real‐time continuous flow ion sensing at electroactive, receptive molecular films, as representatively illustrated by the sensing of bisulfate, dihydrogen phosphate and chloride at electro‐active halogen bonding and hydrogen bonding ferrocenyl anion receptive SAMs. This was achieved by repeat SWV cycling of the sensor and an automated analysis of the obtained voltammograms with a peak‐fitting algorithm. This data analysis methodology enabled facile signal generation and monitoring of anion levels under flow over multiple hours with an analytical performance that is similar, or better, than under standard, static conditions. In addition, it is also associated with an over one order of magnitude improvement of the sensors' LODs in comparison to simpler “peak picking” data extraction. The novel flow SWV approach supports sensing with high temporal resolution (≈3.7 s) and may further enable more fundamental studies, such as the determination of interfacial ion binding kinetics.

Importantly, this voltammetric flow methodology is applicable to sensing of any (ionic) analyte that can reversibly induce a voltammetric response of an electro‐active interface and presents an important step in the translation of such sensors from laboratory to real‐life applications.

## Conflict of interest

The authors declare no conflict of interest.

## Supporting information

As a service to our authors and readers, this journal provides supporting information supplied by the authors. Such materials are peer reviewed and may be re‐organized for online delivery, but are not copy‐edited or typeset. Technical support issues arising from supporting information (other than missing files) should be addressed to the authors.

Supporting Information
