# Supplementary material for: Real‐time Voltammetric Anion Sensing Under Flow
Source: Chemistry. 2021 Oct 27;27(70):17700–6. doi: 10.1002/chem.202103249 (PMC9297856; doi:10.1002/chem.202103249)
Supplement: Supplementary file 1 — Supporting Information [file CHEM-27-17700-s001.pdf]

// Chemistry–A European Journal

Supporting Information

Real-time Voltammetric Anion Sensing Under Flow

Sophie C. Patrick, Robert Hein, Mohamed Sharafeldin, Xiaoxiong Li, Paul D. Beer, and Jason J. Davis*

Table of Contents:

S1 Materials and Experimental Details 1

S1.1 General Information 1

S1.2 Electrochemical Measurements & SAM Formation 1

S1.3 Voltammetric Measurements 1

S1.4 Binding Isotherm Analysis and LOD Determination 2

S2 Voltammetry of 1.XB/HB$_{SAM}$ in the Presence of Acid 3

S3 Microfluidic Continuous Flow Experiments 6

S3.1 Continuous Voltammetric Measurements 6

S3.2 3D printed Microfluidic Cells 6

S3.3 Flow Rate Dependence & Controls 10

S3.4 Optimisation of SWV Parameters for Flow Measurements 11

S3.5 Current Response of Long-term Flow Experiment 12

S4 Data Analysis 13

S4.1 AsymFit Method 13

S4.2 MATLAB Code 16

S5 Analytical Performance Under Continuous Flow 18

S6 Tabulated Raw Data 21

S7 References 24

S1 Materials and Experimental Details

S1.1 General Information

All experiments were performed at room temperature in the presence of oxygen. All commercially available chemicals and solvents were used as received without further purification. All hygroscopic tetrabutylammonium (TBA) salts were stored in vacuum desiccators at room temperature. Ultrapure water was obtained from a Milli-Q system (18.2 MΩcm). Supporting electrolyte ($TBAClO_4$ from Sigma Aldrich) was of electrochemical grade. Receptors **1.XB/HB** were synthesised as described previously.[1]

S1.2 Electrochemical Measurements & SAM Formation

All experiments were conducted using an Autolab Potentiostat (Metrohm) or PalmSens4 Potentiostat with a three-electrode setup equipped with a gold disc working electrode (BaSi, 1.6 mm diameter) and platinum wire counter electrode. A non-aqueous Ag|$AgNO_3$ reference electrode (with an inner filling solution of 10 mM $AgNO_3$, 100 mM $TBAClO_4$ in ACN) was utilised for all experiments and all potentials are reported wrt. to this reference electrode. All experiments were carried out with 100 mM $TBAClO_4$ as a supporting electrolyte with additional 10 mM $HClO_4$, as indicated. In all cases, including sensing studies, the ionic strength was maintained at a constant 100 mM TBA-anion (+ 10 mM $HClO_4$) throughout.

Au disc electrodes were cleaned according to previously reported protocols.[2] Immediately following the cleaning procedure, the Au disc electrodes were rinsed thoroughly with water and ethanol and immersed in a solution of 0.25 mM **1.XB/HB** in ACN overnight in the dark. Subsequently, the Au disc electrodes were rinsed with copious amounts of ACN and then used immediately. Detailed surface characterisation of the so-formed SAMs is reported elsewhere.[1]

S1.3 Voltammetric Measurements

Static square wave voltammetry (SWV) measurements were conducted with a step potential of 2 mV, amplitude of 20 mV and frequency of 25 Hz. Continuous flow SWV measurements were carried out between -0.25 to 0.3 V with a step potential of 2 or 5 mV (typically 5 mV,

unless otherwise indicated), amplitude of 20 mV and frequency of 50 Hz, with all pre/post-equilibration times set to 0 s.

S1.4 Binding Isotherm Analysis and LOD Determination

All anion-induced shifts are reported with respect to the potential in the baseline preceding each injection for flow experiments or with respect to the initial SWV preceding the first addition for static titrations. All binding isotherm fitting was carried out with OriginPro 2017. All binding constants are rounded to three significant figures and were obtained by fitting of the sensing isotherms to the Langmuir-Freundlich model (Eqn. 1). For quantitative analysis, the sensing isotherms for $H_2PO_4^-$ were corrected for full protonation of $H_2PO_4^-$ by the acidified electrolyte, by correcting isotherms by -10 mM.

LODs were calculated according to Eqn. S1 where σ is the standard deviation of the baseline/blank and S is the slope of the linear region of the sensor response (herein also referred to as "sensitivity"). For continuous voltammetric measurements under flow, σ was determined from the root-mean-square deviation of a linear fit of ten data points ($E_{1/2}$ values) in the initial baseline of the sensograms (determined by the respective analysis method as used for the measurement, PeakPick or AsymFit), immediately preceding the response to the first addition of analyte. For static voltammetric measurements, σ was determined as the standard deviation of ten data points ($E_{1/2}$ values) from ten SWVs performed immediately preceding the first analyte addition. S was determined from the slope of a linear fit to the pseudo-linear regime of each respective binding isotherm, with a range between 0-11 mM for HSO_4^- and Cl^-, and either 9.5-13.4 or 10.5-13.4 mM for $H_2PO_4^-$ (for static and continuous measurements, respectively).

$$\boldsymbol{LOD} = \frac{3\sigma}{S} \qquad \text{Eqn. S1}$$

Experimental protocols for flow measurements are detailed in Section S3.

S2 Voltammetry of 1.XB/HB$_{SAM}$ in the Presence of Acid

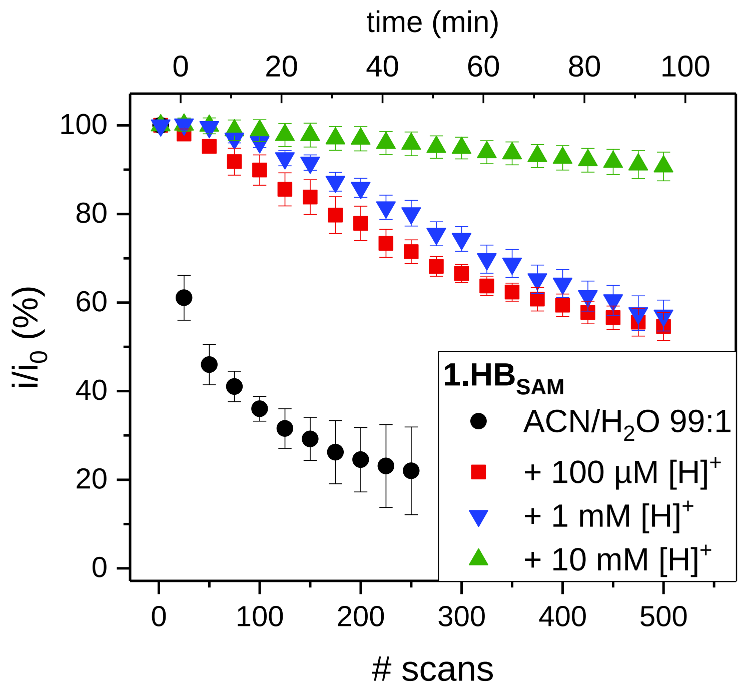

Figure S2.1. Redox stability, as assessed by the relative decrease in peak currents (i/i_0), of **1.HB$_{SAM}$** upon repeated cycling in ACN/H_2O 99:1, 100 mM $TBAClO_4$ in the presence of various concentrations of $HClO_4$. The measurements in the absence of acid (black circles) were stopped after 250 scans.

As alluded to in the main text, acidification of the electrolyte by $HClO_4$ affects the sensor response towards different anions differently (by protonation of the anion, whose conjugate acid does not bind to the receptors) and is dependent on the acid concentration and the basicity of the anion (see Figures S2.2 and S2.3). The specific degree of protonation of these anions is difficult to assess in the electrolyte system (ACN/H_2O 99:1); the well-known acidity trends in water ($HClO_4$ > HCl > H_2SO_4 > H_3PO_4) do not directly translate to a (mixed) organic/aqueous solvent system. For example, in pure ACN the following trend would be expected: $HClO_4$ > HBr > H_2SO_4 > HNO_3 > HCl.[3] In this mixed electrolyte it can be assumed that the added $HClO_4$ is the strongest acid, capable of almost complete protonation of $H_2PO_4^-$ where only a small/negligible response at concentrations $[A^-] < [H^+]$ is observed (Figures S2.2C and S2.3C). In contrast, the less basic Cl^-, and HSO_4^- remain deprotonated to a more significant degree with a smaller, yet still significant response at $[A^-] < [H^+]$ (Figures S2.2A-B and S2.3A-B).

However, as the response patterns of both **1.XB/HB$_{SAM}$** in the presence of various acid concentrations are identical it can be concluded that the acid only influences the solution-phase composition (i.e. the degree of anion protonation) and does not directly influence the binding/response behaviour of the films.

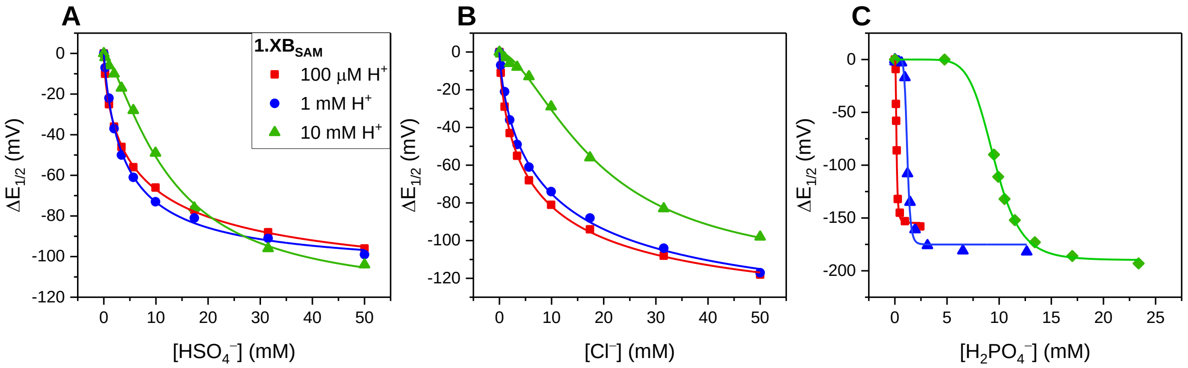

Figure S2.2. Cathodic voltammetric shifts of **1.XB$_{SAM}$** upon titration with (A) HSO_4^- (B) Cl^- and (C) $H_2PO_4^-$ in ACN/H_2O 99:1 and various concentrations of acid. Lines represent fits to the Langmuir-Freundlich model (Eqn. 1).

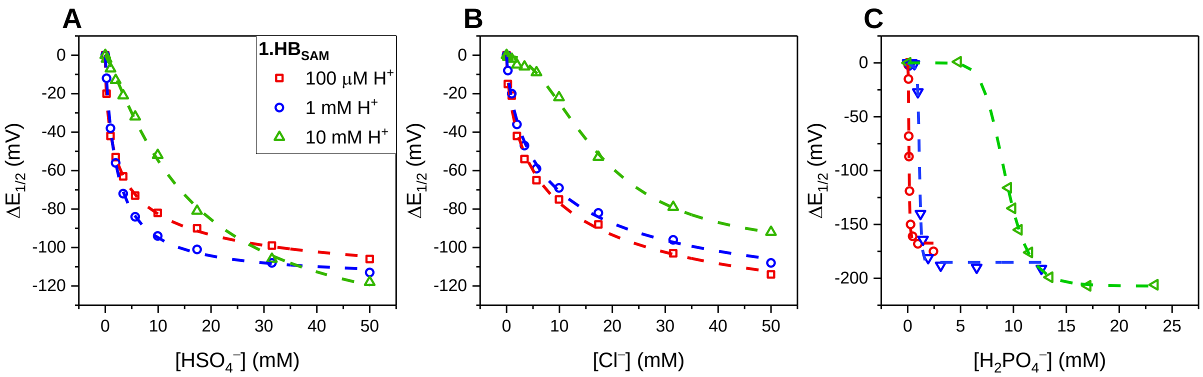

Figure S2.3. Cathodic voltammetric shifts of **1.HB$_{SAM}$** upon titration with (A) HSO_4^- (B) Cl^- and (C) $H_2PO_4^-$ in ACN/H_2O 99:1 and various concentrations of acid. Lines represent fits to the Langmuir-Freundlich model (Eqn. 1).

In the presence of 10 mM $HClO_4$ the binding isotherms for all anions are affected to such a degree that standard binding models cannot accurately describe them anymore. As the pK_a of the anions in the solvent system are not known, the concentration of free anion cannot be calculated. Consequently, no corrections (or adjusted binding models) can be applied.

We thus utilise the Langmuir-Freundlich isotherm as a semi-empirical model to describe the sensor response. "n" is typically interpreted as a heterogeneity factor and herein accounts for the "heterogeneity" imposed by partial anion protonation.

Eqn. 1 was chosen as an empirical model which relates the shift in potential (ΔE) to coverage, whereby the maximum shift in potential induced (ΔE_{max}) by specific target binding correlates to a maximum coverage (θ = 1). Additionally, it is able to account for some of the inhomogeneities observed, that alternative, simpler models (e.g. Langmuir, Nernstian) cannot.

$$\theta = \frac{(K_{app} * [A^-])^n}{1 + (K_{app} * [A^-])^n} \qquad \text{Eqn. 1}$$

Note when n = 1, this model simplifies to the Langmuir model.

In the case of the basic phosphate it can be assumed that full protonation occurs until $[A^-] = [H^+]$ such that at concentrations of $[A^-] > [H^+]$ no acid is present and the subsequent response obeys standard binding models. In this case the binding isotherm is largely identical to that obtained in the absence of acid but is shifted (i.e. offset) by $\approx [H^+]$.

For sake of simplicity and comparisons with the other anions we herein still utilise Eqn. 1 to describe the $H_2PO_4^-$ sensing isotherms. Nevertheless, for quantitative analyses according to this model (i.e. to obtain K_{app}), all phosphate isotherms were corrected by -10 mM phosphate prior to fitting (see Figure S5.3). Similarly, the LODs given for $H_2PO_4^-$ are "apparent" LODs in the "absence" of acid, i.e. are also corrected by -10 mM.

S3 Microfluidic Continuous Flow Experiments

S3.1 Continuous Voltammetric Measurements

A 3D printed microfluidic cell (see Figures S3.1-S3.2) was utilised for continuous flow experiments. Electrolyte (ACN/H_2O 99:1, 100 mM $TBAClO_4$, 10 mM $HClO_4$) was continuously pushed through the cell by a syringe pump at a flow rate of 500 µL min^{-1} (unless otherwise stated). Integrated into the flow line was an injector system (Rheodyne® Model 9725) through which the analyte solutions (of identical ionic strength, and acid concentration in the same solvent) were injected into the continuous flow (Figure S3.3). The majority of tubing used was Cole-Parmer PEEK tubing other than a small connecting section from the main tubing line to the cell, which was Cole-Parmer MasterFlex Peristaltic tubing.

S3.2 3D printed Microfluidic Cells

All microfluidic cells were produced with an Elegoo Mars 3D printer or a FormLabs Form 2 3D printer using FormLabs Tough 2000 resin, which is chemically resistant to a range of organic solvents. General designs were produced with Autodesk Fusion 360 (see Figure S3.2 for annotated blueprint of design), which were then rendered into compatible (printable) designs in CHITUBOX. A Pt wire (counter electrode) was held in place in a channel adjacent to the main chamber with epoxy resin (Araldite Rapid), and the non-aqueous Ag|$AgNO_3$ reference electrode and Au disc electrode functionalised with **1.XB/HB** were inserted before each experiment, giving an airtight cell with an approximate inner chamber volume of 100 µL.

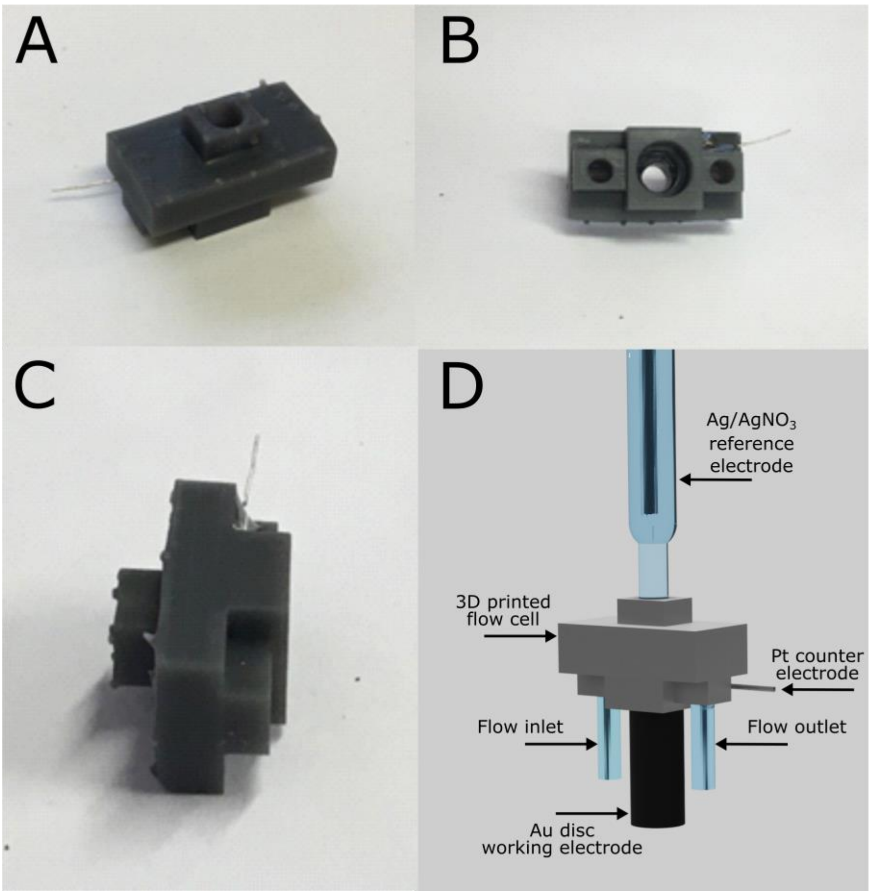

Figure S3.1. A-C) Photos and D) schematic of 3D-printed microfluidic cell.

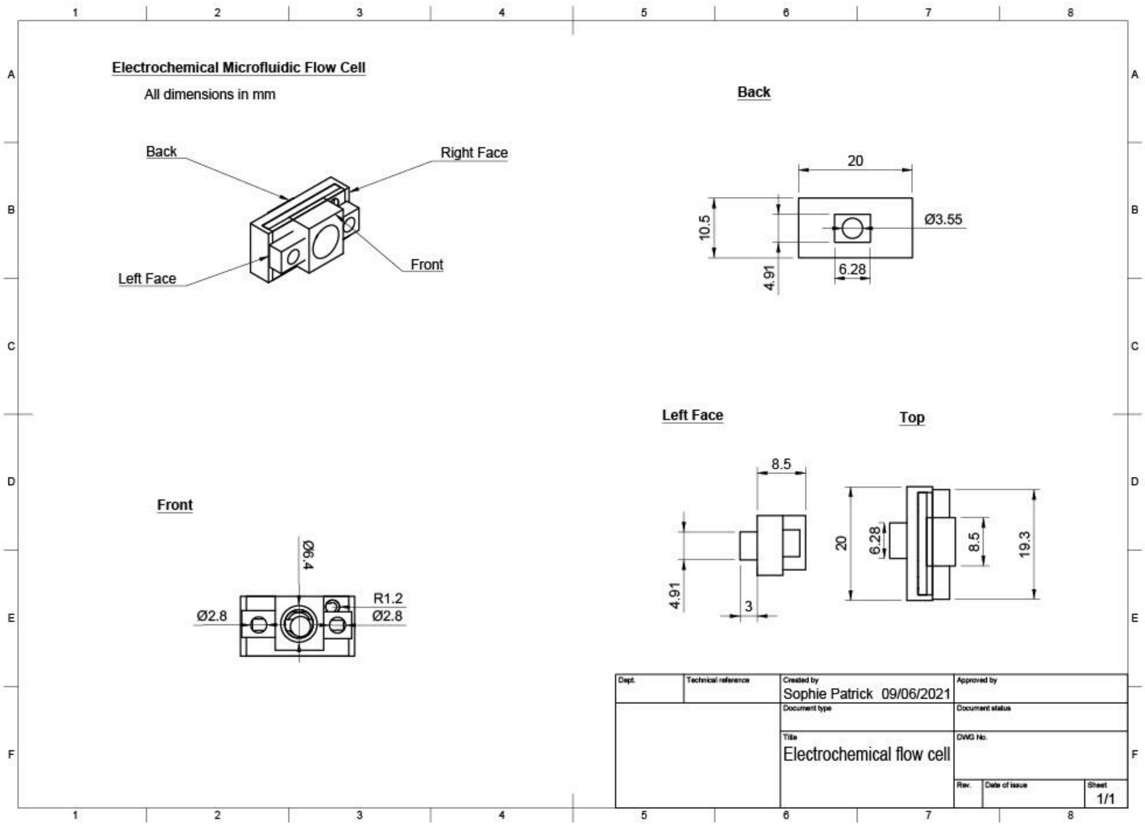

Figure S3.2. Blueprint of 3D-printed microfluidic cell displaying all relevant dimensions in mm. R = radius, Ø = diameter.

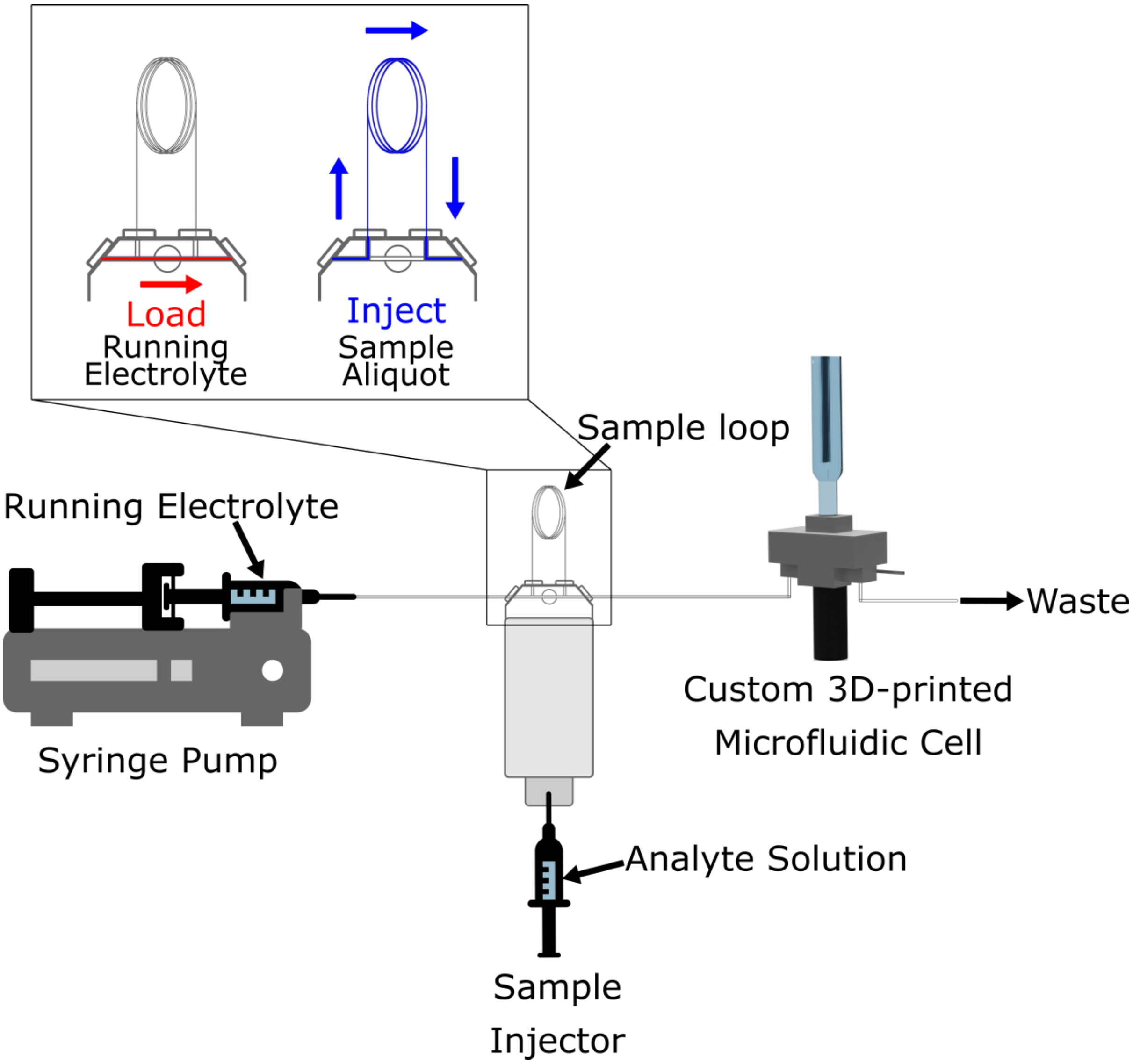

Figure S3.3. Schematic of microfluidic set-up. Inset depicts the two running modes for the microfluidic system: load and inject. Running electrolyte is continuously pumped throughout. When in load mode, only fresh running electrolyte passes through the system but when switched to inject mode, electrolyte passes through the sample loop which was filled with the analyte solution prior to switching.

S3.3 Flow Rate Dependence & Controls

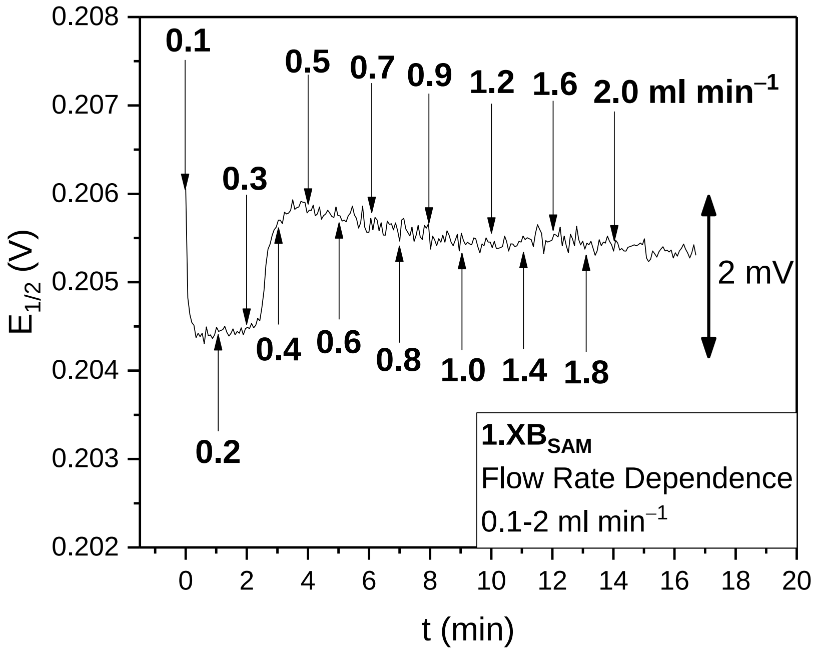

Figure S3.4. Dependence of $E_{1/2}$ of **1.XB$_{SAM}$** on flow rate, over a range of 100 to 2000 µl min^{-1}. Arrow indicates that the total potential fluctuation is within ±2 mV.

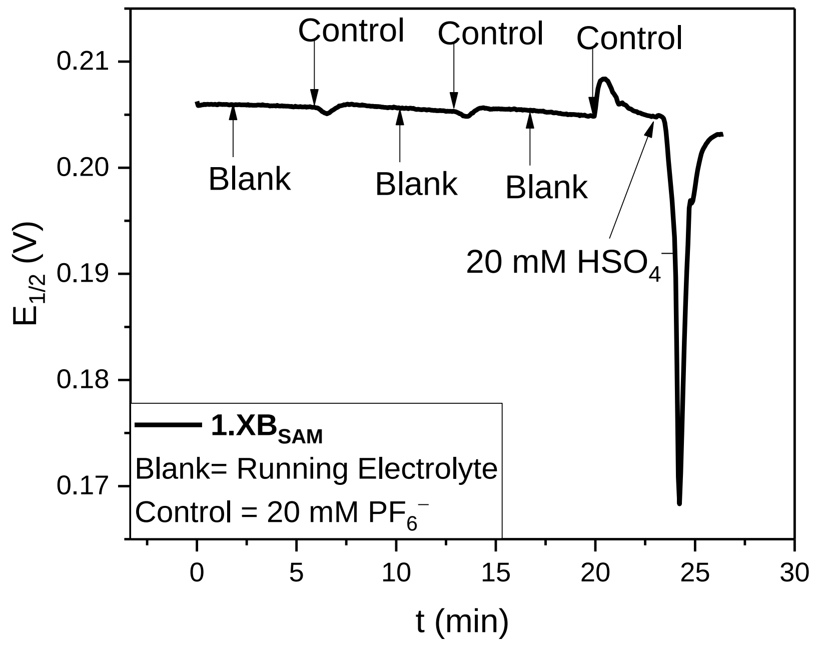

Figure S3.5. Voltammetric response of **1.XB$_{SAM}$** towards blank samples (running electrolyte, 100 mM $TBAClO_4$), controls (20 mM $TBAPF_6$) and 20 mM HSO_4^- under continuous electrolyte flow, flow rate = 500 µL min^{-1}. Each spike represents the response towards aliquots (V_{Sample} = 0.5 mL) of the blanks, controls or HSO_4^- as indicated, showing a negligible response to both the blank and control samples but a significant response to HSO_4^-.

S3.4 Optimisation of SWV Parameters for Flow Measurements

To reduce the time between each SW voltammogram, all pre/post-equilibration times were set to 0 s. A SW amplitude of 20 mV was used throughout; variation in applied amplitude does not affect measuring time. We initially conducted the SWV experiments under identical conditions as typically employed for standard, static titrations, i.e. with a step potential (E_{step}) of 2 mV, an amplitude of 20 mV and a frequency (f) of 25 Hz over a potential range (E_{range}) of 550 mV (–0.25 – 0.3 V).

With these parameters a single SW scan took ≈12.6 s to record, which, under the experimental flow conditions represents a temporal resolution that is slightly too low to accurately measure the anion specific cathodic shifts (see Figure S3.6). With this in mind, we optimised the SWV parameters in order to reduce t_{scan}, which is theoretically given by Eqn. 2. However, in reality a SWV takes somewhat longer to record, as empirically represented by Eqn. S2, where c is a constant, instrument-dependent delay upon repeat SWV cycling (≈1 s with the hardware used herein).

Specifically, f was increased to its hardware limit of 50 Hz, thereby approximately halving t_{scan} to ≈7.2 s. Similarly, E_{step} was increased from 2 to 5 mV, affording a further reduction in t_{scan} to ≈3.7 s. Note that the sensing isotherms are completely identical for E_{step} = 2 or 5 mV (when data analysis is carried out with the AsymFit approach).

$$t_{scan} = \frac{E_{range}}{f \times E_{step}} \qquad \text{Eqn. 2}$$

$$t_{scan} = \frac{E_{range}}{f \times E_{step}} + c \qquad \text{Eqn. S2}$$

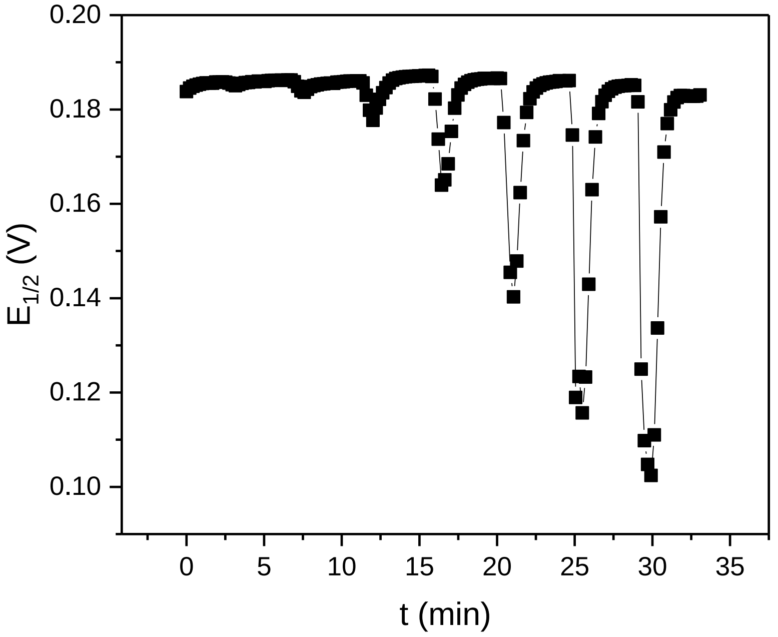

Figure S3.6. Sensogram afforded by an initial titration experiment under continuous flow (f = 25 Hz, E_{step} = 2 mV) shown for the response of **1.XB$_{SAM}$** to increasing concentrations of HSO_4^- (up to 50 mM) in ACN/H_2O 99:1 (100 mM $TBAClO_4$, 10 mM $HClO_4$). Notably, as a result of the comparably low temporal resolution of ≈12.6 s not many data points fall within each response peak arising from analyte addition, such that errors will be larger.

S3.5 Current Response of Long-term Flow Experiment

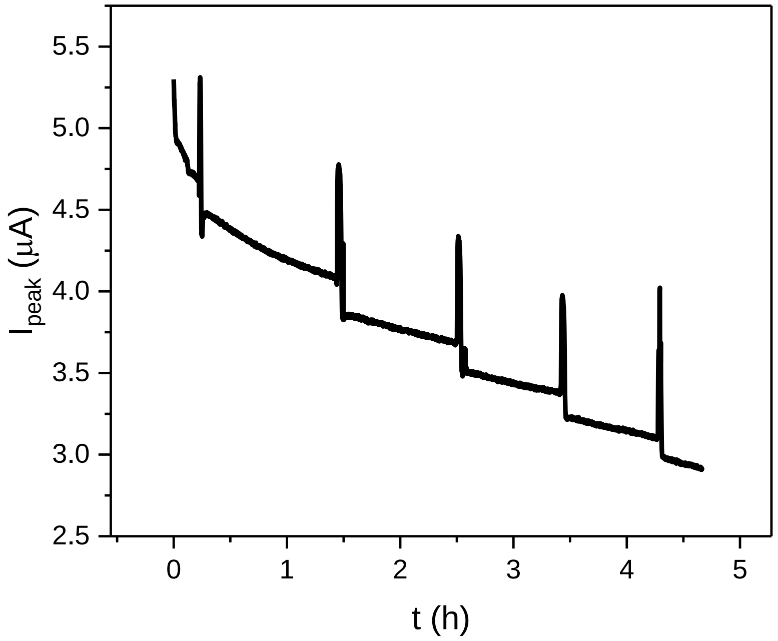

Figure S3.7. Current response of **1.XB$_{SAM}$** in response to five additions of 20 mM of HSO_4^- over 4.5 h, analysed with the AsymFit method.

S4 Data Analysis

S4.1 AsymFit Method

Initialisation parameters for each variable in Eqn. S3 were estimated for each SWV as follows:

- y_0 (alpha), is the initial current value from the isolated data set around each peak.
- A (beta), is the peak height given by the difference between the maximum current and y_0.
- x_c (Guessxpeak) is the estimated peak potential, as determined via the PeakPick method.
- c_1, c_2 and c_3 (full width at half maximum, FWHM) are all given by an estimation for the full width half maximum value, here as the difference between the potential at the 6th and 16th data points from the isolated data set around each peak.

Function (f3):

$$\boldsymbol{y} = \boldsymbol{y_0} + \boldsymbol{A}\left[\frac{\mathbf{1}}{\left(\mathbf{1}+\boldsymbol{e}^{\left(-\left(x-x_c+\frac{c_1}{2}\right)/c_2\right)}\right)} \times \left(\mathbf{1} - \frac{\mathbf{1}}{\left(\mathbf{1}+\boldsymbol{e}^{\left(-\left(x-x_c-\frac{c_1}{2}\right)/c_3\right)}\right)}\right)\right] \qquad \text{Eqn. S3}$$

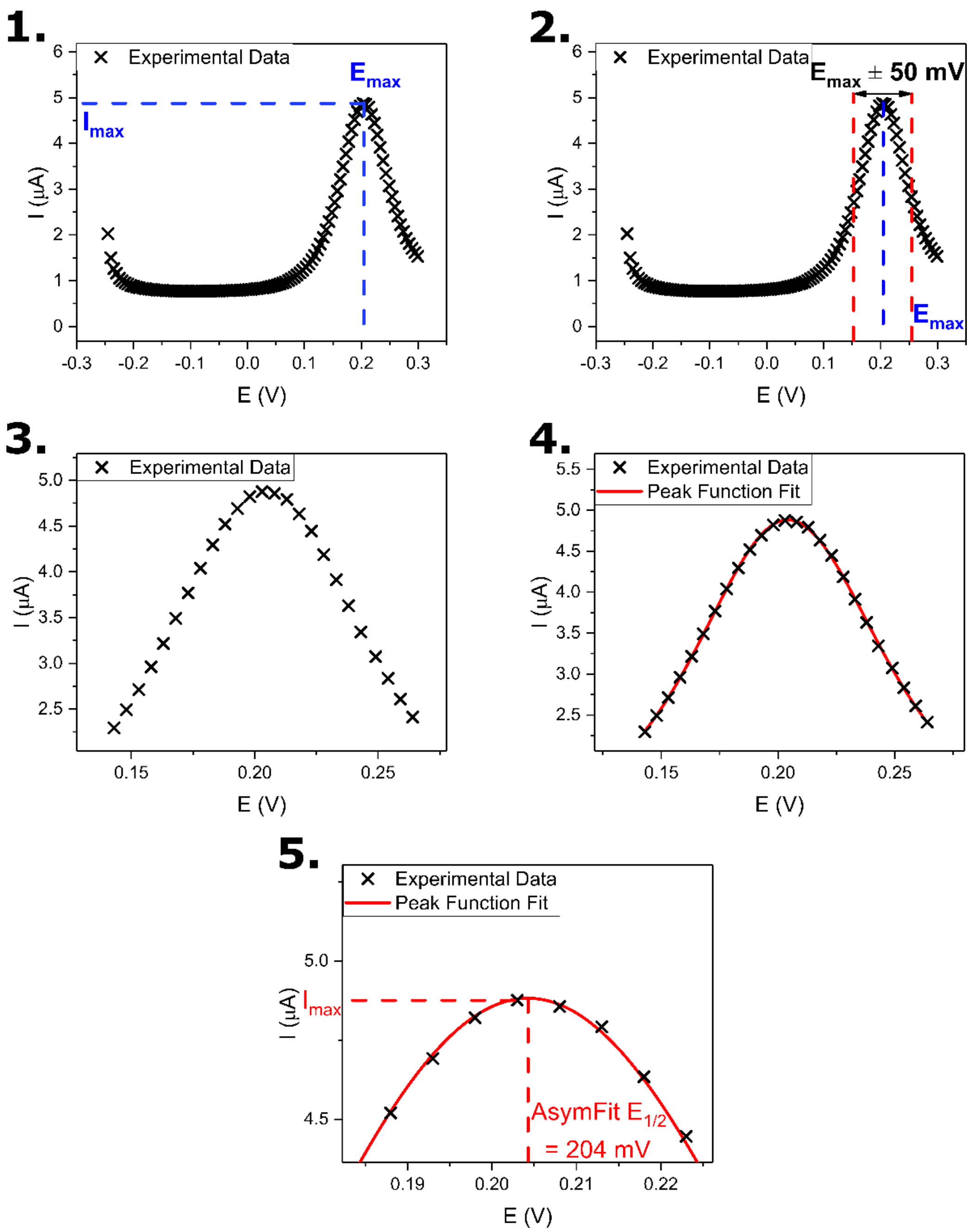

Figure S4.1. Schematic depiction of the steps for the AsymFit data analysis method. 1. An initial estimate for $E_{1/2}$ was obtained from the PeakPick method of the raw data. 2. and 3. All data points ±50 mV around this estimated value were isolated (i.e. the baseline was removed). 4. The isolated peak was fitted according to the asymmetric double sigmoidal function (Eqn. S3). 5. The $E_{1/2}$ of this fitted, continuous peak distribution was obtained by the PeakPick method (as $E_{1/2}$ at I_{max}).

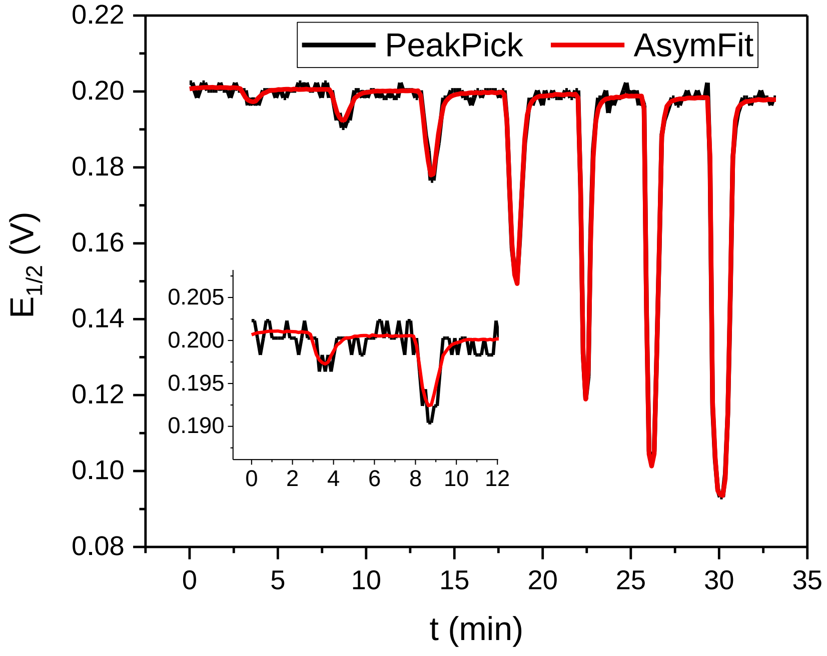

Figure S4.2. Comparison of continuous SWV sensograms for **1.XB$_{SAM}$** in response to increasing concentrations of HSO_4^- up to 50 mM (E_{step}= 2 mV) analysed via the PeakPick method (black line) and AsymFit method (red line). Inset displays the improved fit with the AsymFit method for the first two additions (0.5 and 1.5 mM HSO_4^-).

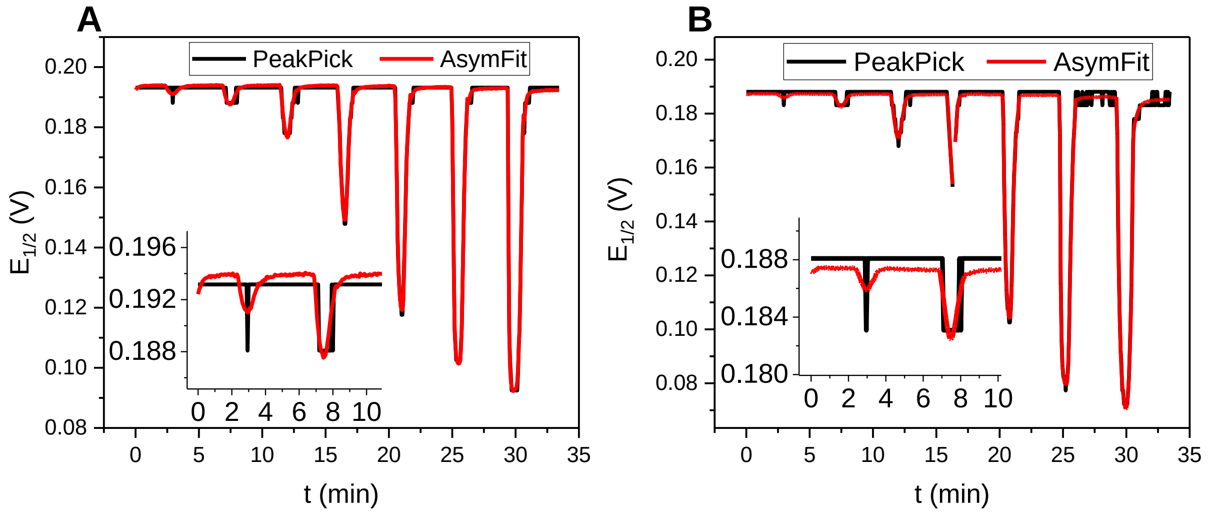

Figure S4.3. Comparison of continuous SWV sensograms for **1.XB$_{SAM}$** in response to increasing concentrations of HSO_4^- up to 50 mM (E_{step}= 5 mV) analysed via the PeakPick method (black line) and AsymFit method (red line). Inset displays the improved fit with the AsymFit method for the first two additions (0.5 and 1.5 mM HSO_4^-). The figures show examples where PeakPick A) overestimates and B) underestimates $E_{1/2}$.

S4.2 MATLAB Code

PeakPick Code:

```
result = [];
AllPeaks = [];
AllPeaks2 = [];
%For loop to apply analysis to n SWVs
for v = 1:n
    i = (2*v) - 1;

    % Extracting the data from the table into variables
    x1 = DataTable{i,:};
    y1 = DataTable{i+1,:};

    % Extracting E and I data for each SWV into separate table
    PeakTable1 = [x1(:),y1(:)];
    % Finding Emax values from maximum I value
    [~,x1] = max(PeakTable1(:,2));
    Peak1 = PeakTable1(x1,:);
    %Combining all Emax values into one table as final result
    AllPeaks = [AllPeaks; Peak1];
```

AsymFit Code:

(run in addition to the PeakPick Code above)

```
%Taking values for I
Y = DataTable{i+1,:};

%Identify maximum I
maximum = max(Y);

%Find location of maximum I in matrix
[a,b] = find(Y==maximum);

%Cut main table down to ±50 mV either side of Emax for each SWV trace
CutTable = DataTable([i:i+1],[(b-12):(b+12)]);

    % Extracting the data from the table into new variables
    x = CutTable{1,:};
    y = CutTable{2,:};

%Curve Fit
%Initialisation parameters
    Guessxpeak = Peak1(1,1);
    alpha = y(1,1);
    beta = maximum-Peak1(1,1);
    fwhm = x(1,16) - x(1,6);

  x0 = [alpha beta Guessxpeak fwhm fwhm fwhm];

%Curve fitting options
options = optimoptions('lsqcurvefit','Algorithm','levenberg-
marquardt','MaxIterations',10000,'MaxFunctionEvaluations',20000,'FunctionTo
lerance',1e-9,'StepTolerance',1e-10,'FiniteDifferenceType','central');
lb = [0 0 0 0 0 0];
ub = [];
```

```
constant = lsqcurvefit(@f4,x0,x,y,lb,ub,options);

%Defining each constant to be optimised
y0 = constant(1);
A = constant(2);
xc = constant(3);
c1 = constant(4);
c2 = constant(5);
c3 = constant(6);

%Extracting final results into one table
result = [result; y0, A, xc, c1, c2, c3];

xmin = min(x);
xmax = max(x);

%Variables of Asymfit
xfit = xmin:0.00001:xmax;
yfit = f3(constant,xfit);

    % Extracting Efit and Ifit data for each SWV into separate table
    PeakTable2 = [xfit(:),yfit(:)];
    % Finding peak values from maximum Ifit value
    [~,xfit] = max(PeakTable2(:,2));
    Peak2 = PeakTable2(xfit,:);
    %Combining all E1/2 values into one table as final result
    AllPeaks2 = [AllPeaks2; Peak2];


end
AllPeaks
AllPeaks2
result
```

S5 Analytical Performance Under Continuous Flow

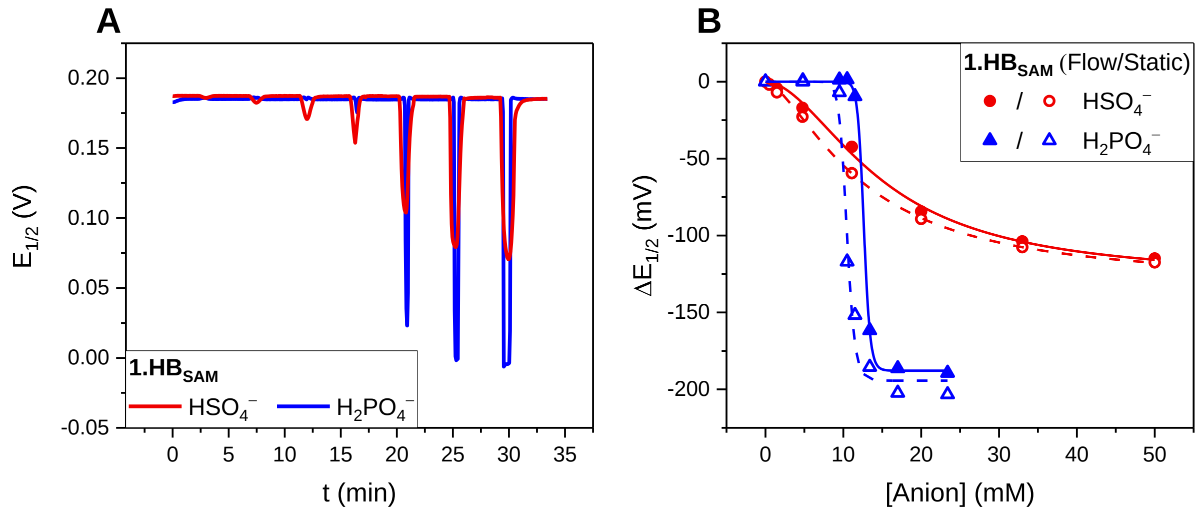

Figure S5.1. (A) Sensograms of $\mathbf{1.HB_{SAM}}$ in response to increasing concentrations of HSO_4^- (red) and $H_2PO_4^-$ (blue), analysed with the AsymFit method. B) Comparison of static (empty symbols) and continuous titrations (filled symbols) with $\mathbf{1.HB_{SAM}}$ in response to HSO_4^- (red circles) and $H_2PO_4^-$ (blue triangles). All isotherms were fitted to the Langmuir-Freundlich model (Eqn. 1).

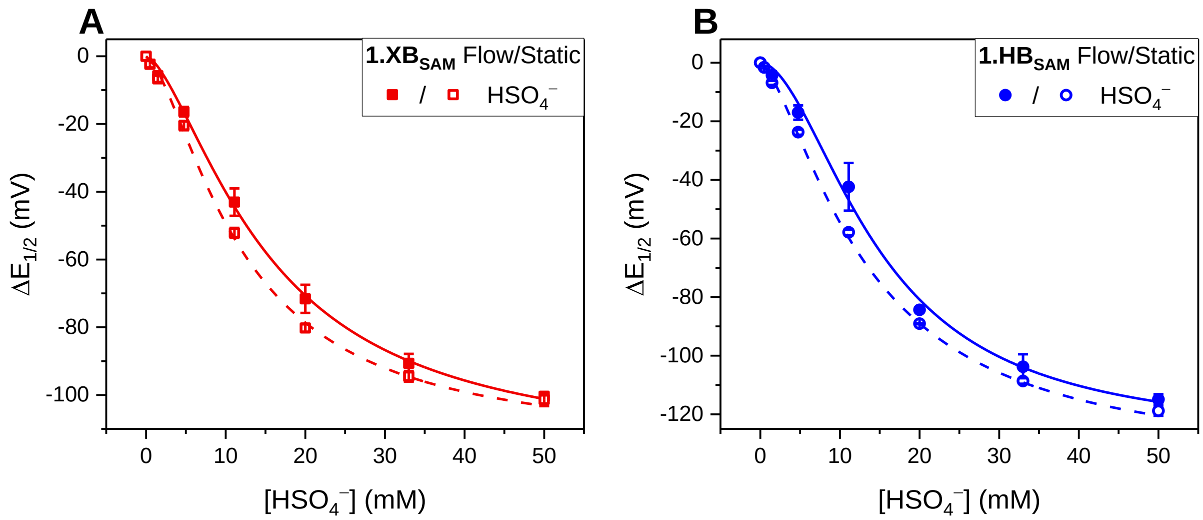

Figure S5.2. Comparison of static (empty symbols) and continuous isotherms (filled symbols) of A) $\mathbf{1.XB_{SAM}}$ and B) $\mathbf{1.HB_{SAM}}$ in response to increasing concentrations of HSO_4^- up to 50 mM. All isotherms were fitted to the Langmuir-Freundlich model (Eqn. 1). Error bars represent one standard deviation of triplicate independent measurements.

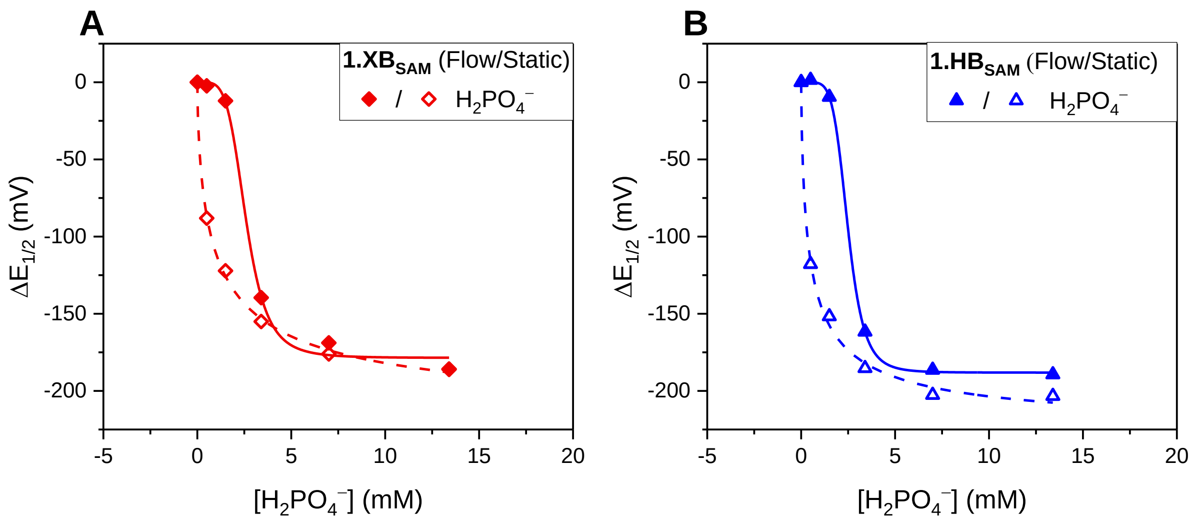

Figure S5.3. Comparison of corrected static (empty symbols) and continuous isotherms (filled symbols) of A) **1.XB$_{SAM}$** and B) **1. HB$_{SAM}$** in response to $H_2PO_4^-$ up to 23.4 mM. All isotherms were fitted to the Langmuir-Freundlich model (Eqn. 1), and were corrected by -10 mM. The uncorrected isotherms are shown in Figures S5.2B and Figure 7B.

As a result of the inherent electroactivity of the Cl^- anion all sensing studies were restricted to a somewhat lower concentration range (up to 33 mM Cl^-) as at higher concentrations the Fc and Cl^- redox processes overlap too significantly to reliably determine the $E_{1/2}$ of the Fc/Fc$^+$ couple.

As a result of the washing steps following each addition whereby the baseline is re-established, minor baseline drifts can be accounted for, which is not possible for static titrations. This may explain why the static and continuous isotherms differ slightly in some cases, in particular for chloride (see Figure S5.5).

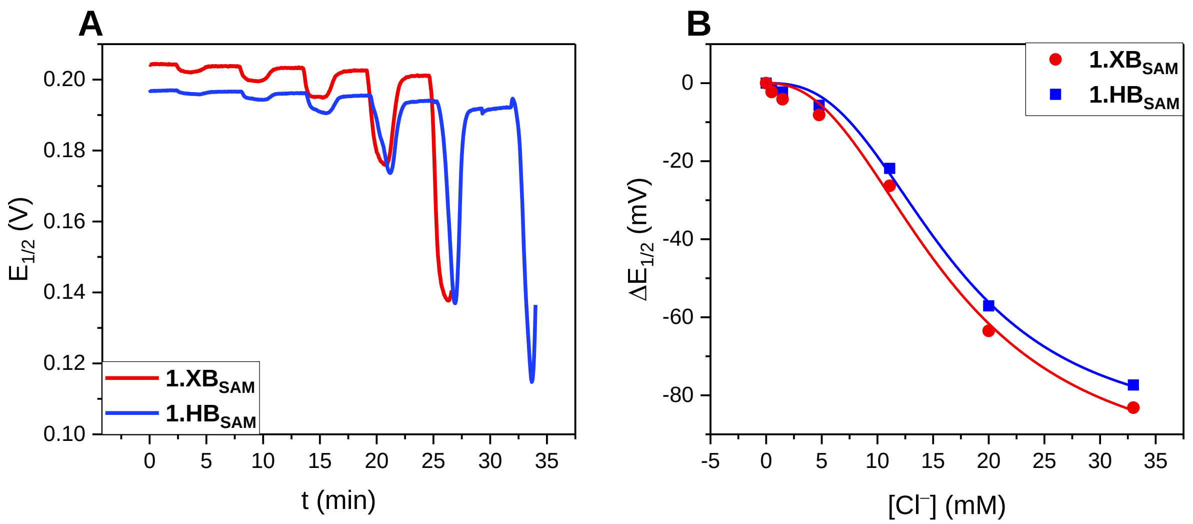

Figure S5.4. (A) Sensograms of **1.XB/HB$_{SAM}$** in response to increasing concentrations of Cl^- (0.5-33 mM, V_{sample}= 1 mL), analysed with the AsymFit method and B) corresponding isotherms. All isotherms were fitted to the Langmuir-Freundlich model (Eqn. 1). The redox activity of Cl^- high concentrations interfered with the AsymFit method applied, therefore data is shown for the SWVs for which the code was successful up to the point of failure.

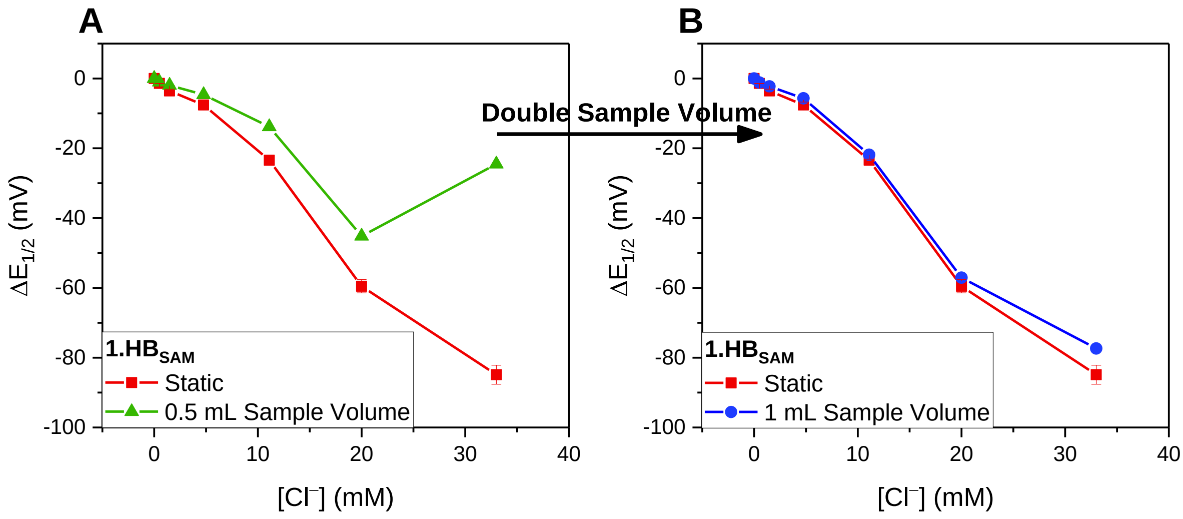

Figure S5.5. Comparison of static (red squares) and continuous titrations (A) green triangles for 0.5 mL aliquot volumes and (B) blue circles for measurements with 1 mL aliquot volumes with **1.HB$_{SAM}$** in response to Cl^-. Connecting lines are to guide the eye only. B) Shows that doubling the aliquot volume to 1 mL was sufficient time to reach the static, equilibrium response.

S6 Tabulated Raw Data

Static AsymFit: Langmuir-Freundlich Fitting Data

Table S1. Apparent binding constants (K_{app}), heterogeneity factors (n) and R^2 values from Langmuir-Freundlich fitting for sensing studies with **1.XB/HB$_{SAM}$** under static conditions in response to HSO_4^-, $H_2PO_4^-$ and Cl^-, which were analysed via the AsymFit method. All errors are mathematical errors from fitting.

	Notes	1.XB/HB	K_{app} (M^{-1})	n	R^2
**HSO_4^-**		XB	91.7 ± 4.0	1.72 ± 0.10	0.999
		HB	80.8 ± 2.3	1.58 ± 0.05	0.999
	Repeat	XB	82.3 ± 7.0	1.49 ± 0.13	0.998
	Repeat	HB	74.1 ± 3.4	1.43 ± 0.06	0.999
	Repeat	XB	81.0 ± 4.6	1.64 ± 0.11	0.999
	Repeat	HB	70.9 ± 3.7	1.48 ± 0.07	0.999
**$H_2PO_4^-$**	Full range up to 23.4 mM	XB	93.1 ± 1.7	15.1 ± 4.0	0.979
	Full range up to 23.4 mM	HB	95.7 ± 1.4	20.4 ± 5.5	0.981
	Corrected by -10 mM	XB	989 ± 224	0.648 ± 0.087	0.999
	Corrected by -10 mM	HB	2120 ± 427	0.708 ± 0.176	0.997
**Cl^-**	Up to 33 mM	XB	52.9 ± 7.7	1.97 ± 0.27	0.997
	Up to 33 mM	HB	54.3 ± 5.8	2.37 ± 0.32	0.997

Static AsymFit: LODs and Sensitivities

Table S2. Sensitivities (slope of linear region at low concentrations, including R^2), baseline standard deviations and limits of detection (LODs) for sensing studies with **1.XB/HB$_{SAM}$** under static conditions, which were analysed via the AsymFit method. All errors are mathematical errors from fitting.

	Linear Range (mM)	1.XB/HB	Sensitivity (mV mM^{-1})	R^2	Baseline s.d. (mV)	LOD (µM)
**HSO_4^-**	0-11	XB	4.81 ± 0.14	0.997	0.0916	57.2
	0-11	HB	5.35 ± 0.12	0.998	0.110	61.7
	0-11	XB	4.72 ± 0.08	0.999	0.162	103
	0-11	HB	5.29 ± 0.03	0.999	0.0763	43.2
	0-11	XB	4.51 ± 0.13	0.998	0.0641	42.7
	0-11	HB	5.13 ± 0.08	0.999	0.0492	28.8
**$H_2PO_4^-$**	9.5-13.4	XB	36.0 ± 10.3	0.859	0.0906	20.1
	9.5-13.4	HB	41.8 ± 14.4	0.808	0.0479	8.58
**Cl^-**	0-11	XB	2.71 ± 0.25	0.975	0.189	210
	0-11	HB	2.08 ± 0.19	0.975	0.135	195

Flow AsymFit (5 mV): Langmuir-Freundlich Fitting Data

Table S3. Apparent binding constants (K_{app}), heterogeneity factors (n) and R^2 values from Langmuir-Freundlich fitting for sensing studies with **1.XB/HB$_{SAM}$** under continuous flow conditions (E_{step} = 5 mV) in response to HSO_4^-, $H_2PO_4^-$ and Cl^-, which were analysed via the AsymFit method. All errors are mathematical errors from fitting.

	Notes	1.XB/HB	K_{app} (M^{-1})	n	R^2
**HSO_4^-**		XB	73.0 ± 4.3	1.60 ± 0.10	0.999
		XB	69.8 ± 6.1	1.56 ± 0.14	0.998
	Repeat	XB	53.6 ± 4.5	1.49 ± 0.10	0.999
	Repeat	HB	69.6 ± 2.8	1.55 ± 0.06	0.999
	Repeat	HB	70.5 ± 6.1	2.07 ± 0.29	0.995
	Repeat	HB	63.7 ± 7.0	2.26 ± 0.44	0.991
**$H_2PO_4^-$**	Full range up to 23.4 mM	XB	78.5 ± 0.7	25.5 ± 3.2	0.997
	Full range up to 23.4 mM	HB	79.0 ± 0.2	31.0 ± 1.1	0.999
	Corrected by -10 mM	XB	385 ± 20	4.63 ± 0.72	0.997
	Corrected by -10 mM	HB	401 ± 5	5.74 ± 0.19	0.999
**Cl^-**	Up to 33 mM	XB	61.2 ± 8.1	2.35 ± 0.44	0.994
	Up to 33 mM	HB	60.4 ± 4.4	2.65 ± 0.33	0.997

Flow AsymFit (5 mV): LODs and Sensitivities

Table S4. Sensitivities (slope of linear region at low concentrations, including R^2), baseline standard deviations and limits of detection (LODs) for sensing studies with **1.XB/HB$_{SAM}$** under continuous flow conditions (E_{step} = 5 mV), which were analysed via the AsymFit method. All errors are mathematical errors from fitting.

	Linear Range (mM)	1.XB/HB	Sensitivity (mV mM^{-1})	R^2	Baseline s.d. (mV)	LOD (µM)
**HSO_4^-**	0-11	XB	4.21 ± 0.15	0.996	0.0917	65.4
	0-11	XB	3.96 ± 0.12	0.997	0.0717	54.3
	0-11	XB	3.33 ± 0.11	0.997	0.0617	55.5
	0-11	HB	4.80 ± 0.13	0.998	0.0903	56.4
	0-11	HB	3.71 ± 0.21	0.991	0.0204	16.5
	0-11	HB	3.02 ± 0.12	0.995	0.0322	32.0
**$H_2PO_4^-$**	10.5-13.4	XB	49.9 ± 14.5	0.922	0.0628	3.77
	10.5-13.4	HB	59.3 ± 17.1	0.923	0.0177	0.894
**Cl^-**	0-11	XB	2.27 ± 0.20	0.977	0.0678	89.6
	0-11	HB	1.93 ± 0.20	0.968	0.0308	48.0

Flow AsymFit (2 mV): Langmuir-Freundlich Fitting Data

Table S5. Apparent binding constants (K_{app}), heterogeneity factors (n) and R^2 values from Langmuir-Freundlich fitting for sensing studies with **$1.XB_{SAM}$** under continuous flow conditions (E_{step} = 2 mV) in response to HSO_4^-, $H_2PO_4^-$ and Cl^-, which were analysed via the AsymFit method. All errors are mathematical errors from fitting.

	Notes	1.XB	K_{app} (M^{-1})	n	R^2
**HSO_4^-**		XB	74.2 ± 8.0	1.43 ± 0.14	0.998
	Repeat	XB	57.5 ± 5.7	1.69 ± 0.17	0.998
	Repeat	XB	88.4 ± 6.3	1.59 ± 0.13	0.998

Flow AsymFit (2 mV): LODs and Sensitivities

Table S6. Sensitivities (slope of linear region at low concentrations, including R^2), baseline standard deviations and limits of detection (LODs) for sensing studies with **$1.XB_{SAM}$** under continuous flow conditions (E_{step} = 2 mV), which were analysed via the AsymFit method. All errors are mathematical errors from fitting.

	Linear Range (mM)	1.XB	Sensitivity (mV mM^{-1})	R^2	Baseline s.d. (mV)	LOD (µM)
**HSO_4^-**	0-11	XB	4.46 ± 0.08	0.999	0.0431	29.0
	0-11	XB	3.21 ± 0.06	0.999	0.0922	86.1
	0-11	XB	4.81 ± 0.04	0.999	0.0738	46.0

Peak Pick (2 mV): Langmuir-Freundlich Fitting Data

Table S7. Apparent binding constants (K_{app}), heterogeneity factors (n) and R^2 values from Langmuir-Freundlich fitting for sensing studies with **$1.XB_{SAM}$** under continuous flow conditions (E_{step} = 2 mV) in response to HSO_4^-, $H_2PO_4^-$ and Cl^-, which were analysed via the PeakPick method. All errors are mathematical errors from fitting.

	Notes	1.XB	K_{app} (M^{-1})	n	R^2
**HSO_4^-**		XB	65.8 ± 10.4	1.25 ± 0.13	0.997
	Repeat	XB	57.5 ± 5.7	1.69 ± 0.17	0.998
	Repeat	XB	86.9 ± 6.4	1.49 ± 0.11	0.998

Peak Pick (2 mV): LODs and Sensitivities

Table S8. Sensitivities (slope of linear region at low concentrations, including R^2), baseline standard deviations and limits of detection (LODs) for sensing studies with **1.XB/HB$_{SAM}$** under continuous flow conditions (E_{step} = 2 mV), which were analysed via the PeakPick method. All errors are mathematical errors from fitting.

	**Linear Range (mM)**	**1.XB**	**Sensitivity (mV mM^{-1})**	**R^2**	**Baseline s.d. (mV)**	**LOD (µM)**
**HSO_4^-**	0-11	XB	4.36 ± 0.17	0.995	1.19	819
	0-11	XB	3.25 ± 0.13	0.995	0.895	825
	0-11	XB	4.83 ± 0.07	0.999	1.02	633

S7 References

1. Hein, R.; Li, X.; Beer, P. D.; Davis, J. J. Enhanced voltammetric anion sensing at halogen and hydrogen bonding ferrocenyl SAMs. *Chem. Sci.* **2021,** *12*, 2433-2440.
2. Patrick, S. C.; Hein, R.; Docker, A.; Beer, P. D.; Davis, J. J. Solvent Effects in Halogen and Hydrogen Bonding Mediated Electrochemical Anion Sensing in Aqueous Solution and at Interfaces. *Chem. Eur. J.* **2021,** *27*, 10201-10209.
3. Kütt, A.; Rodima, T.; Saame, J.; Raamat, E.; Mäemets, V.; Kaljurand, I.; Koppel, I. A.; Garlyauskayte, R. Y.; Yagupolskii, Y. L.; Yagupolskii, L. M.; Bernhardt, E.; Willner, H.; Leito, I. Equilibrium Acidities of Superacids. *J. Org. Chem.* **2011,** *76*, 391-395.
